# A molecular insight into the dissociable regulation of associative learning and motivation by the synaptic protein neuroligin-1

**DOI:** 10.1186/s12915-020-00848-7

**Published:** 2020-09-14

**Authors:** Jiaqi Luo, Jessica M. Tan, Jess Nithianantharajah

**Affiliations:** Florey Institute of Neuroscience and Mental Health, Florey Department of Neuroscience, Melbourne Brain Centre, University of Melbourne, 30 Royal Parade, Parkville, Victoria 3052 Australia

**Keywords:** Cost-benefit trade-off, Learning and memory, Motivation, NL1, Response latency, Response vigor, Rodent touchscreens

## Abstract

**Background:**

In a changing environment, a challenge for the brain is to flexibly guide adaptive behavior towards survival. Complex behavior and the underlying neural computations emerge from the structural components of the brain across many levels: circuits, cells, and ultimately the signaling complex of proteins at synapses. In line with this logic, dynamic modification of synaptic strength or synaptic plasticity is widely considered the cellular level implementation for adaptive behavior such as learning and memory. Predominantly expressed at excitatory synapses, the postsynaptic cell-adhesion molecule neuroligin-1 (Nlgn1) forms trans-synaptic complexes with presynaptic neurexins. Extensive evidence supports that Nlgn1 is essential for NMDA receptor transmission and long-term potentiation (LTP), both of which are putative synaptic mechanisms underlying learning and memory. Here, employing a comprehensive battery of touchscreen-based cognitive assays, we asked whether impaired NMDA receptor transmission and LTP in mice lacking Nlgn1 does in fact disrupt decision-making. To this end, we addressed two key decision problems: (i) the ability to learn and exploit the associative structure of the environment and (ii) balancing the trade-off between potential rewards and costs, or positive and negative utilities of available actions.

**Results:**

We found that the capacity to acquire complex associative structures and adjust learned associations was intact. However, loss of Nlgn1 alters motivation leading to a *reduced* willingness to overcome effort cost for reward and an *increased* willingness to exert effort to escape an aversive situation. We suggest Nlgn1 may be important for balancing the weighting on positive and negative utilities in reward-cost trade-off.

**Conclusions:**

Our findings update canonical views of this key synaptic molecule in behavior and suggest Nlgn1 may be essential for regulating distinct cognitive processes underlying action selection. Our data demonstrate that learning and motivational computations can be dissociated within the same animal model, from a detailed behavioral dissection. Further, these results highlight the complexities in mapping synaptic mechanisms to their behavioral consequences, and the future challenge to elucidate how complex behavior emerges through different levels of neural hardware.

## Background

In a changing environment, a challenge for the brain is to adaptively guide behavior towards survival which involves the processing of sensory information, selecting between actions that will most likely result in a beneficial outcome, and executing these actions. These complex cognitive abilities emerge from the physical architecture of the brain: from circuits to neurons, synapses, and ultimately the molecular components that comprise the protein signaling complexes at synaptic terminals. This hardware forms the basis to enable signaling and plasticity within and between brain regions, thus supporting the emergence of behavior. In line with this logic, intact synaptic transmission and plasticity are widely held as the key cellular level mechanisms required for adaptive behavior such as learning and memory. At the postsynaptic density (PSD) of excitatory synapses, neuroligin-1 (Nlgn1) is a cell-adhesion molecule that binds presynaptic neurexins to form trans-synaptic complexes [1, 2]. Aligning PSD components with presynaptic neurotransmitter release sites [3–5], Nlgn1 directly binds postsynaptic scaffolds including PSD-95 [3] and promotes retention of α-amino-3-hydroxyl-5-methyl-4-isoxazole-propionate (AMPA) and *N*-methyl-d-aspartate (NMDA) receptors by indirect intracellular and direct extracellular interactions in developing and mature synapses [6–9].

Importantly, Nlgn1 is well established to be required for intact NMDA receptor function and long-term potentiation (LTP), both of which are the key synaptic mechanisms thought to underlie learning and memory [10–13]. NMDA receptor-mediated postsynaptic currents have been consistently shown to be decreased across several brain regions by Nlgn1 knockout or knockdown [7, 14–23] and, conversely, increased by Nlgn1 overexpression [22–24]. Further, Nlgn1 has been repeatedly shown to be essential for synaptic plasticity and the induction of both NMDA receptor-dependent and independent LTP in multiple brain regions [7, 14, 15, 17–20, 25, 26]. Therefore, a reasonable assumption may be that Nlgn1 is also important for learning and memory processes. Indeed, Blundell and colleagues reported disrupted grooming behavior and spatial learning and memory in the Morris water maze in mice lacking Nlgn1 [14]. This is further supported by impaired performance in the water maze in Nlgn1 overexpression mouse models [24, 27] and contextual and cued fear memory recall deficits in rats with basal lateral amygdala knockdown of Nlgn1 [19], collectively supporting the idea that Nlgn1 is necessary for learning and memory. In contrast to the extensive molecular and physiological characterization of Nlgn1, only these few studies have examined Nlgn1 in behavior, and specifically none has evaluated different components of complex cognitive behavior in detail. Thus, whether impaired NMDA receptor function and synaptic plasticity caused by loss of Nlgn1 translates to impact all forms of learning and the wider cognitive repertoire is unclear.

In this study, employing a comprehensive battery of touchscreen-based cognitive assays, we sought to assess male and female mice lacking Nlgn1 on two key decision problems: (1) the ability to learn and exploit the associative structure of the environment and (2) balancing the trade-off between potential rewards and costs, or positive and negative utilities associated with available actions. We found that mice lacking Nlgn1 have an intact capacity to acquire complex associative structures and adjust learned associations. However, loss of Nlgn1 alters motivation leading to a *reduced* willingness to overcome response effort for reward and *increased* willingness to exert effort to escape an aversive situation. We suggest these divergent phenotypes may converge on a model of increased weighting on negative utilities, highlighting a novel valence-dependent role of Nlgn1 in balancing the weighting on positive and negative utilities in reward-cost trade-off. Our data identify unexpected findings that update current views of this key synaptic molecule to show Nlgn1 is essential for regulating distinct cognitive processes underlying decision-making. Our findings demonstrate that learning and motivational computations can be behaviorally dissected, contributing to unraveling the genetic architecture of dissociable cognitive modules. Further, these behavioral findings highlight the complexity in directly mapping synaptic mechanisms to their behavioral consequences, thus the future challenge and importance of elucidating how complex behavior emerges through different levels of neural hardware.

## Results

### Nlgn1 is not essential for learning complex associative structures

Intact long-term forms of plasticity and NMDA receptor function are both synaptic mechanisms thought to be required for learning and memory (e.g., [13, 28–35]). Based on the established impairments in NMDA receptor function and LTP combined with the previous behavioral reports, we hypothesized that Nlgn1 is likely to be important for acquiring associative structures of the environment, and using these structures to optimize action selection. To address this, we assessed male and female null mutant mice lacking Nlgn1 (*Nlgn1*^−/−^) and control wildtype (WT) littermates in a series of rodent touchscreen cognitive tests, where mice were required to make responses via nose-pokes to different visual stimuli displayed on a touchscreen to obtain rewards under different test situations. Of note, across all the cognitive tests and analyses performed, we observed no significant interactions between genotype and sex (except for that presented in Fig. 4f, data in Additional file 1: Fig. S13); therefore, combined data for both sexes by genotype will be presented for clarity. Additionally, we employed trial-by-trial analyses that better describe complex behavioral data (see the “Methods” section and Additional file 2: Table S1 for detailed statistical results).

**Fig. 1:**
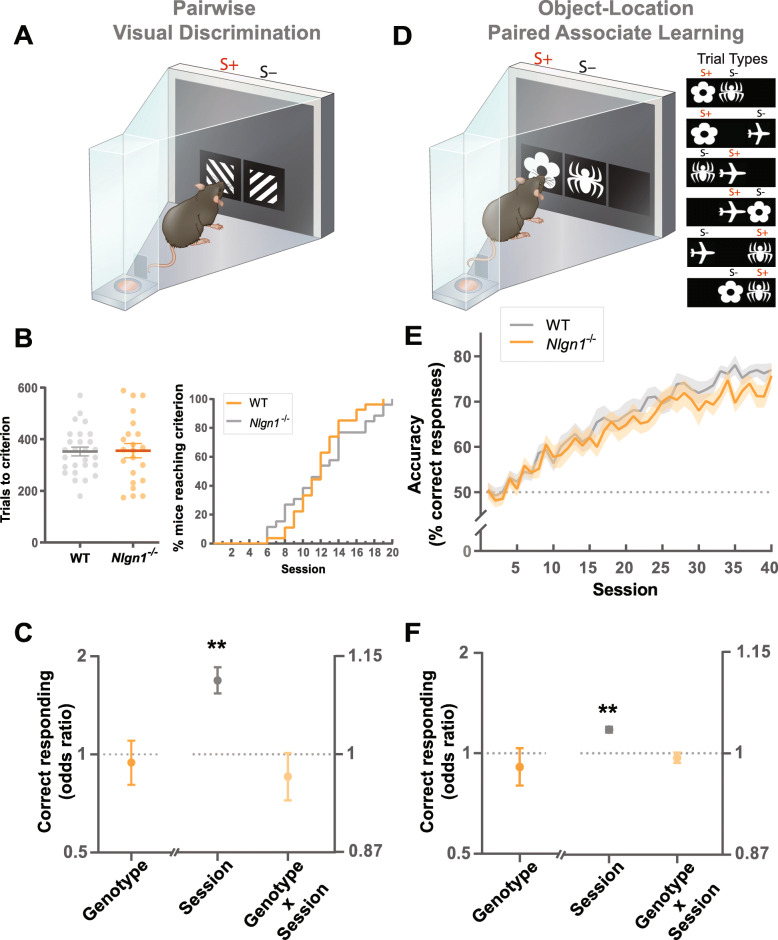
*Nlgn1*^−/−^ mice display normal associative learning. **a**–**c** Pairwise visual discrimination learning task. **a** Stimuli used, S+ (rewarded stimulus), S−(unrewarded stimulus). **b** Number of trials to reach learning criterion; two-way ANOVA, values represent means ± SEM. Percentage (%) of mice reaching learning criterion across sessions. **c** Effect of genotype, session, and their interaction on correct responding, GLLAMM (logistic link function), **p < 0.005 significantly different from 1, values represent the estimated effect of the variables on the odds of correct responding (odds ratio) ± 95% CI. **d**–**f** Object-location paired associate learning task. **d** Visual stimuli and their paired correct locations (6 possible trial types). **e** Visuospatial learning curve showing performance accuracy across sessions, values represent means ± SEM. **f** Effect of genotype, session, and their interaction on correct responding, GLLAMM (logistic link function), **p < 0.005 significantly different from 1, values represent odds ratio ± 95% CI

Animals first underwent several phases of instrumental training (touchscreen pre-training) to learn to initiate the commencement of trials and selectively nose-poke simple visual stimuli displayed on the touchscreen in order to obtain a liquid reward (strawberry milk) [36, 37]. *Nlgn1*^−/−^ and WT mice required similar numbers of sessions to complete the pre-training phases, indicating loss of Nlgn1 does not impact the acquisition of simple instrumental conditioning (Additional file 1: Fig. S2). Following pre-training, mice were introduced to the pairwise visual discrimination task, a forced choice paradigm where two visually similar stimuli were presented pseudorandomly between two locations (left or right side of the touchscreen). Responses to one of the stimuli were rewarded (S+) while responses to the other were unrewarded (S−) (Fig. 1a). The visual discrimination task therefore requires mice to learn to perceptually discriminate the stimuli and selectively respond to the correct or rewarded stimulus regardless of stimulus location. Performance accuracy (percentage of correct responses) was the primary measure used to track learning across training sessions, until a learning criterion was reached. We found that *Nlgn1*^−/−^ mice required similar numbers of trials to reach the discrimination learning criterion as WT controls (Fig. 1b). Similarly, the percentage of *Nlgn1*^−/−^ and WT mice reaching criterion across sessions was not different (Fig. 1b). To understand how key variables including genotype, sex, and session affect response accuracy, we estimated the effect of these variables on trial outcomes (correct/incorrect responses) using a mixed-effect generalized linear model [38]. We observed a highly significant effect of session as expected, reflecting the improvements on response accuracy over sessions. However, there was no effect of genotype on trial outcomes (correct responding of *Nlgn1*^−/−^ relative to WT expressed as odds ratio, Fig. 1c) nor a significant genotype × session interaction, indicating that both *Nlgn1*^−/−^ and WT mice acquired visual discrimination learning at a similar rate (Fig. 1c), consistent with our trials to learning criterion and percentage of mice reaching criterion analyses. Furthermore, these data combined with the normal instrumental conditioning (pre-training) also confirm loss of Nlgn1 does not impair basic perceptual processing abilities.

To increase demands on associative complexity by having to integrate both visual and spatial information as features defining the reward contingencies, we employed the object-location paired associate learning task which requires mice to learn not only the perceptual features of three stimuli (flower, plane, spider) but also their unique rewarded location on the touchscreen (left, center, right respectively). On each trial, only two stimuli are presented: one displayed in its correct location (S+) and the other in an incorrect location (S−) (Fig. 1d). Compared to the visual discrimination task, the greater difficulty in acquiring visuospatial associations in this object-location paired associate learning task is reflected in a slower rate of learning, evident by the increased number of training sessions (Fig. 1e) and smaller effect size of session (Fig. 1f). Despite this increased difficulty, we again observed *Nlgn1*^−/−^ mice were able to acquire the complex object-location associations similar to WT mice (Fig. 1e, f). There were subtle suggestions that *Nlgn1*^−/−^ mice may have reduced accuracy in later sessions (25 onwards), but since there was no significant genotype × session interaction (*p* = 0.1, Additional file 2: Table S1), we deemed it inappropriate to follow up further analyses separating out stages of paired associate learning.

### Flexible updating of learned associations is not altered by loss of Nlgn1

**Fig. 2:**
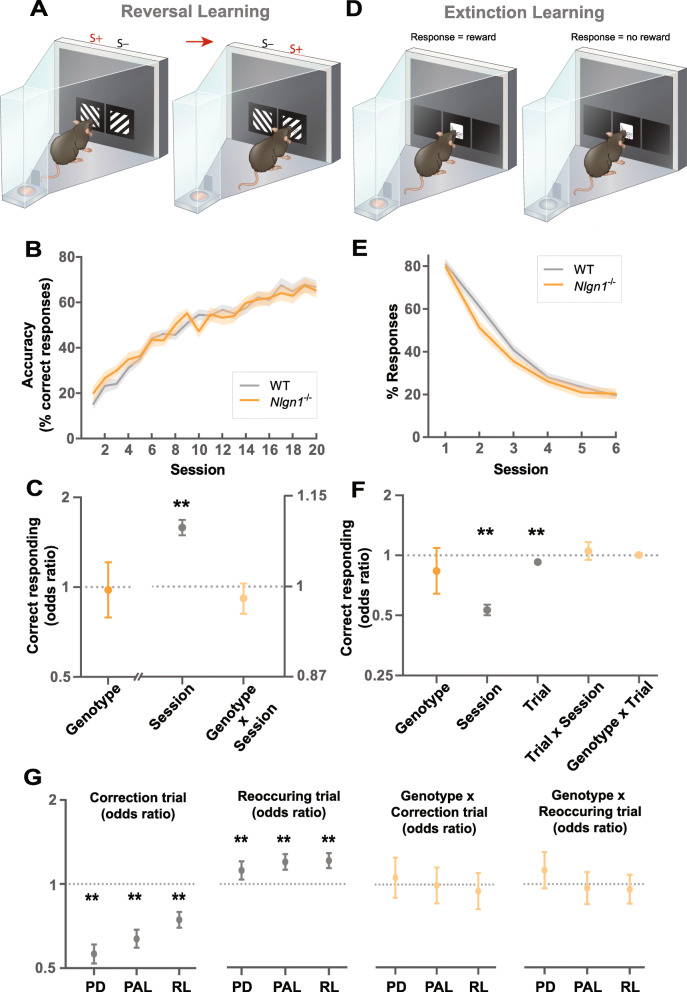
*Nlgn1*^−/−^ mice display normal adjusting of learned associations. **a**–**c** Reversal learning task. **a** Reward contingencies were switched following acquisition of learning criterion for visual discrimination, S+ (rewarded stimulus), S− (unrewarded stimulus). **b** Reversal learning curve showing performance accuracy across sessions, values represent means ± SEM. **c** Effect of genotype, session, and their interaction on correct responding, GLLAMM (logistic link function), **p < 0.005 significantly different from 1, values represent odds ratio ± 95% CI. **d–f** Extinction learning task. **d** Once robust instrumental responding to a performance criterion was reached, responses were no longer rewarded. **e** Extinction learning curve showing percentages of responses across sessions, values represent means ± SEM. **f** Effect of genotype, session, trial within a session, and their interactions on responding, GLLAMM (logistic link function), **p < 0.005 significantly different from 1, values represent the estimated effect of the variables on the odds of correct responding (odds ratio) ± 95% CI. **g** *Nlgn1*^−/−^ and WT mice both display similar levels of perseverative behavior in response selection across tasks (pairwise visual discrimination, PD; object-location paired associate learning, PAL; reversal learning, RL). Mice were less accurate on correction trials (effect of correction trial on correct responding, effect sizes < 1); more accurate on pseudorandom trials with reoccurring stimulus configurations (effect of reoccurring pseudorandom trial on correct responding, effect size > 1); but there were no differences due to genotype (effect of correction trial × genotype interaction, effect of reoccurring pseudorandom trial × genotype interaction). GLLAMM (logistic link function), **p < 0.005, values represent the estimated effect of the variables on the odds of correct responding (odds ratio) ± 95% CI

Adapting to dynamic environments where the outcome of a response is not always stable requires the ability to inhibit learned responses once they no longer yield positive outcomes, and explore alternatives. To examine the requirement of Nlgn1 for flexible adjustment of response selection, we employed two tests: reversal and extinction learning. Both these tests probe flexible responding but can depend on distinct genetic and neural basis (e.g., [39]). First, we examined cognitive flexibility in a test of reversal learning. Once mice had reached the learning criterion on pairwise visual discrimination (Fig. 1a–c), we reversed the reward contingencies so that the previously rewarded stimulus was now unrewarded and vice versa (Fig. 2a). All mice displayed a strong tendency to respond to the previously rewarded stimulus (now S−) at the beginning of reversal learning, then gradually shifted their responding to the updated S+ (previous S−) as expected (Fig. 2b). We observed no differences in response accuracy between *Nlgn1*^−/−^ and WT mice throughout reversal learning (effect of genotype) nor the rate of reversal learning (genotype × session interaction) (Fig. 2c).

Second, we investigated extinction learning measuring the rate at which mice stop making a learned instrumental response when those responses no longer result in an outcome, and there are no competing operant response alternatives. Mice were first trained to robustly respond to a simple stimulus (white square), and once a stable performance criterion was reached, extinction was tested in which responses to the stimulus were no longer rewarded (Fig. 2d). On each trial, mice could either make or omit a response within a set time window. One challenge in quantifying the rate of instrumental extinction is that feedback on reward contingency is only provided after a response. As a result, animals that display slower instrumental extinction for example (therefore made more responses) will have received more learning opportunities due to greater feedback, and vice versa. To minimize potential differences in learning opportunities, we set a limit on the number of trials per session for all animals and assessed extinction learning across sessions (Fig. 2e, Additional file 1: Fig. S3). We observed no difference between *Nlgn1*^−/−^ and WT mice on whether they responded or omitted a trial throughout extinction (effect of genotype) and rates of extinction both within a session (effect of trial, genotype × trial) and across sessions (effect of session, genotype × session) (Fig. 2f). To confirm potential differences in extinction rate was not masked by differences in learning opportunities, we also analyzed the rate of extinction as the effect of cumulative responses and again found no differences (data not shown) indicating intact instrumental extinction learning.

Previous work has reported that *Nlgn1*^−/−^ mice display increased self-grooming [14] thought to represent repetitive and stereotypic behavior common in brain disorders such as autism spectrum disorder and obsessive-compulsive disorder [40, 41]. We were therefore interested to explore measures of repetitive or perseverative response selection across our multiple learning assays. In the pairwise visual discrimination, object-location paired associate learning, and reversal learning tasks, a correct response to a first-presentation pseudorandom trial (referred to as a “trial”) was always followed by another trial, where the stimuli and location are displayed in a pseudorandom and counterbalanced manner. In contrast, an incorrect response was always followed by a “correction trial” where the exact same stimulus-location configuration of that (pseudorandom) trial is repeatedly presented until mice switch their response to make a correct response and earn a reward (Additional file 1: Fig. S4A). A perseveration index (PI) calculated as the average number of correction trials committed per incorrect response has been commonly used to measure perseverative responding (e.g., [35, 36, 42]). However, to more explicitly and quantitatively examine perseverative responding on correction trials, we estimated the effect of correction trials on correct responding. Mice were less accurate on correction trials suggesting a tendency to reselect the same incorrect response previously selected (Fig. 2g, Additional file 1: Fig. S5). Consistent with a tendency of repeating the previous response, mice were more accurate when the same stimulus-location configuration happened to reoccur on a consecutive trial (referred to as a “reoccurring trial”), therefore more likely to reselect a correct response previously selected (Fig. 2g, Additional file 1: Fig. S5). However, we found no differences in perseverative responding between *Nlgn1*^−/−^ and WT mice (genotype × correction trial, genotype × reoccurring trial) (Fig. 2g). These data show mice appear to have a general tendency towards perseverative action selection.

### Mice lacking Nlgn1 take longer to perform instrumental actions for rewards

**Fig. 3:**
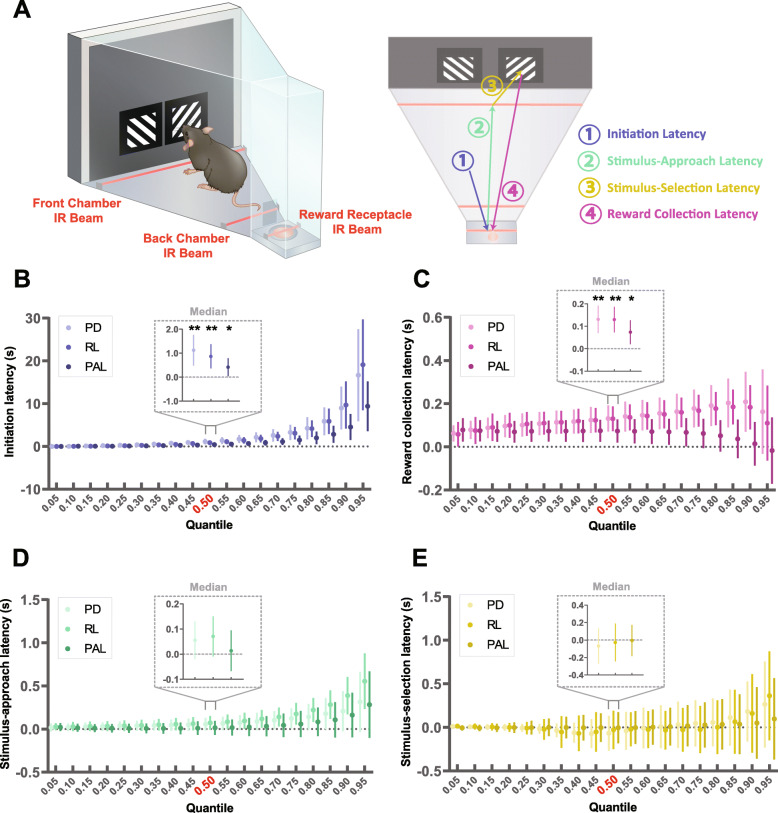
*Nlgn1*^−/−^ take longer to perform instrumental actions for rewards. **a** Infrared (IR) beams located within the chambers at the front, back, and reward receptacle allow the dissection of multiple reaction times (initiation latency; stimulus-approach and stimulus-selection latency; reward collection latency, see the “Methods” section and Additional file 1: Fig. S4). *Nlgn1*^−/−^ mice took longer to **b** initiate trials, **c** collect rewards, and **d** approach the touchscreen (stimulus-approach latency) but not **e** stimulus-selection across tasks (effect of genotype > 0). **b**–**e** Latency differences between *Nlgn1*^−/−^ and WT mice estimated by quantile regression from the 0.05th to 0.95th quantile at steps of 0.05, with insets highlighting median quantile values. Pairwise visual discrimination (PD), reversal learning (RL), object-location paired associate learning (PAL), data arranged in order of task training. *p < 0.05, **p < 0.005, quantile regression values represent estimated latency difference between *Nlgn1*^−/−^ and WT mice ± 95% CI

Decision-making in the natural world involves more than choosing the response with the highest expected rewards. Actions may have very different effort requirements; therefore, balancing the trade-off between rewards and costs is a crucial part of maximizing the net utility of actions. We have so far examined task measures that involve action selection between two alternatives that require the same amount of physical effort and only differ in the expected rewards (e.g., performance accuracy calculated on correct vs incorrect responding). But like most naturalistic decision problems, these touchscreen-based tasks are free-operant tasks in that mice are free to choose a wide range of other actions (e.g., exploring, resting) instead of choosing to execute actions towards earning a reward (initiating a trial, making a response, collecting a reward). To capture an animal’s engagement in performing instrumental actions in our tasks, we analyzed several latency parameters: trial initiation, response, and reward collection (Fig. 3a, see the “Methods” section and Additional file 1: Fig. S4B). Response latency was further separated into( i) stimulus-approach latency: time taken after initiating a trial to reach the front of the chamber near the touchscreen and (ii) stimulus-selection latency: time taken from reaching the front of the chamber near the touchscreen and making a nose-poke response to a stimulus. Dissecting the response latencies revealed stimulus-selection latencies positively predicted performance accuracy suggesting it influences the decision between correct and incorrect responses, whereas stimulus-approach latencies did not, suggesting it reflects the decision between choosing to make a response or not (Additional file 1: Fig. S7). Distribution-wide analysis (0.05–0.95th quantile at 0.05 steps) across all three of our tasks (pairwise visual discrimination, object-location paired associate learning, reversal learning) consistently revealed the same pattern for the various latency measures (Fig. 3). *Nlgn1*^−/−^ mice were significantly slower to initiate trials, approach stimuli, and collect rewards (Fig. 3b–d, Additional file 1: Fig. S8). Although differences in median stimulus-approach latency were not statistically significant between genotypes, distribution-wide quantile regressions showed a clear distributional shift towards longer latencies (Fig. 3d). In comparison, stimulus-selection latencies were nearly identical between *Nlgn1*^−/−^ and WT mice (Fig. 3e).

### Loss of Nlgn1 reduces motivation to overcome response effort for rewards

**Fig. 4:**
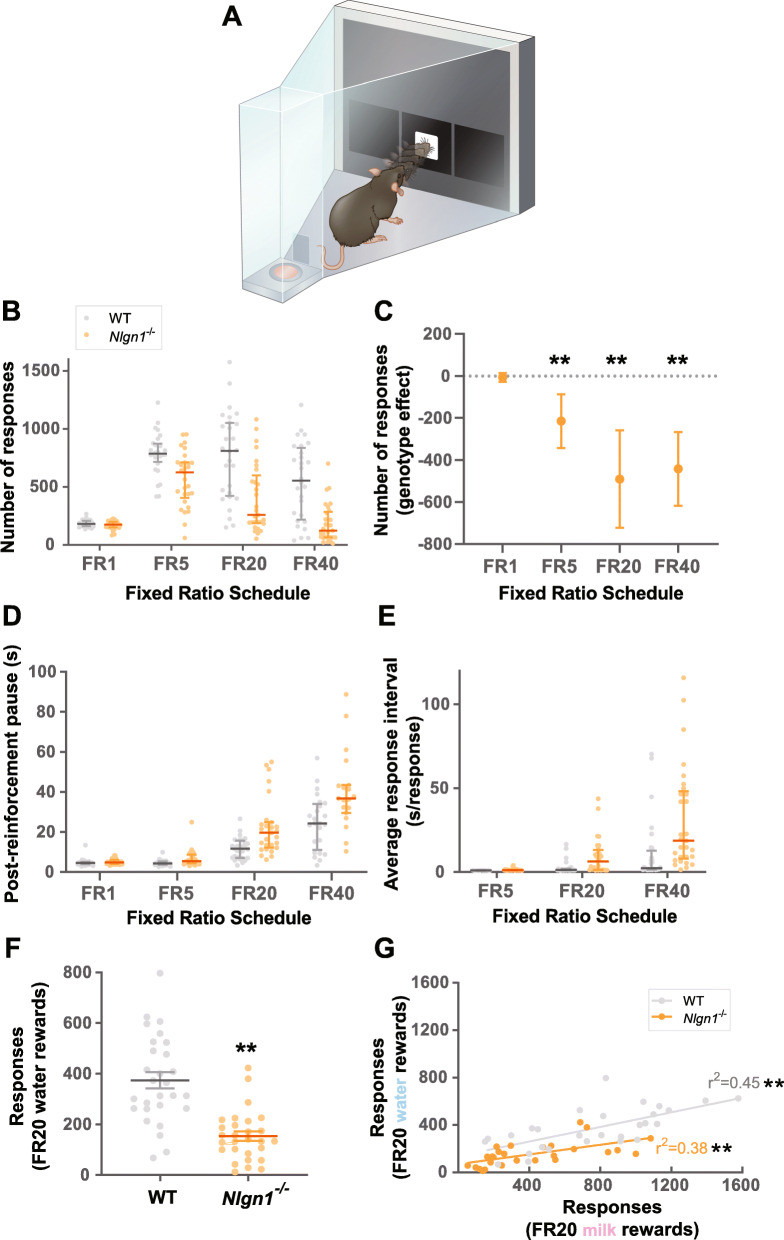
*Nlgn1*^−/−^ mice show reduced motivation to overcome response effort for rewards. **a** Fixed ratio task required a fixed number of nose-pokes (e.g., 5 responses, FR5) for a single reward. Responses to FR1, 5, 20, and 40 were measured. **b** Total number of responses averaged over three sessions per ratio requirement. Bars indicate 1st, 2nd (median), and 3rd quartiles. **c** *Nlgn1*^−/−^ mice made fewer responses in the higher ratio requirements (effect of genotype < 0), quantile regression (median), **p < 0.005, values represent estimated effect of genotype on response counts ± 95% CI. **d** Post-reinforcement pause (time to the first response after consuming a reward, seconds) averaged over three sessions per ratio requirement. **e** Median inter-response interval (time spent per response after an animal made the first response of a trial, seconds per response), averaged across three sessions per ratio requirement. See Additional file 1: Fig. S11 for response-by-response breakdown of raw inter-response intervals. **f** *Nlgn1*^−/−^ mice also made fewer responses for water rewards on fixed ratio 20 (FR20), linear regression, **p < 0.005 values represent means ± SEM. **g** Number of responses made for milk rewards positively correlated with number of responses made for water rewards on FR20 for both WT and *Nlgn1*^−/−^ mice, linear regression, **p < 0.005

Based on the increased latencies *Nlgn1*^−/−^ mice displayed, we hypothesized Nlgn1 may be important for regulating specific components of motivational processing. To examine this, we first tested naive mice sequentially across sessions that required a fixed ratio of responding for rewards with increasing demands (FR1–40, Fig. 4a). We wondered whether loss of Nlgn1 might reduce the number of responses at higher ratio requirements where responding has a lower utility, resembling well-characterized models of amotivation [43–45]. Indeed, when the response-reward ratio was low (FR1), *Nlgn1*^−/−^ mice performed like WT controls indicating similar motivation to respond when response utility is high, and similar rate to reach satiety. However, as this ratio increased (FR5, FR20, FR40), Nlgn*1*^−/−^ mice started to make significantly fewer responses, with the reduction being greater at the higher ratio requirements (Fig. 4b, c, Additional file 1: Fig. S9). This increasing difference in responses between genotypes across ratio requirements was primarily driven by the non-linear increase in the latency taken to re-engage in responding after consuming a reward (post-reinforcement pause, Fig. 4d, Additional file 1: Fig. S10A) and the average time interval between each subsequent response (Fig. 4e, Additional file 1: Fig. S10B, Fig. S11). Next, using a separate naive cohort of mice, we wanted to see if we could observe the same motivational phenotype in a progressive ratio task where ratio requirements progressively increased within a session until mice stop responding (breakpoint). Indeed, we were able to reproduce the same finding with *Nlgn1*^−/−^ mice making fewer responses and therefore having a lower breakpoint relative to controls (Additional file 1: Fig. S12).

Reduced motivation could be due to taste insensitivity to palatable rewards with higher caloric value (e.g., strawberry milk in this case). To examine whether the observed phenotype in *Nlgn1*^−/−^ mice was specific to high-fat-high-sugar strawberry milk rewards, we next used water as the reward and measured responding at a fixed ratio where a robust difference was observed using strawberry milk (FR20). Similar to that observed with strawberry milk rewards, *Nlgn1*^−/−^ mice made significantly fewer responses for water rewards (Fig. 4f), with this effect being stronger in females compared to males (Additional file 1: Fig. S13A). Importantly, there were significant positive correlations between the numbers of responses for strawberry milk and water rewards, indicating that mice (either WT or *Nlgn1*^−/−^) that were more motivated by strawberry milk also responded more for water (Fig. 4g, Additional file 1: Fig. S13B). Together, these data show loss of Nlgn1 impacts instrumental responding motivated by both hunger and thirst.

### *Nlgn1*^−/−^ mice exert *less* effort to earn rewards but *more* effort to escape from aversion

**Fig. 5:**
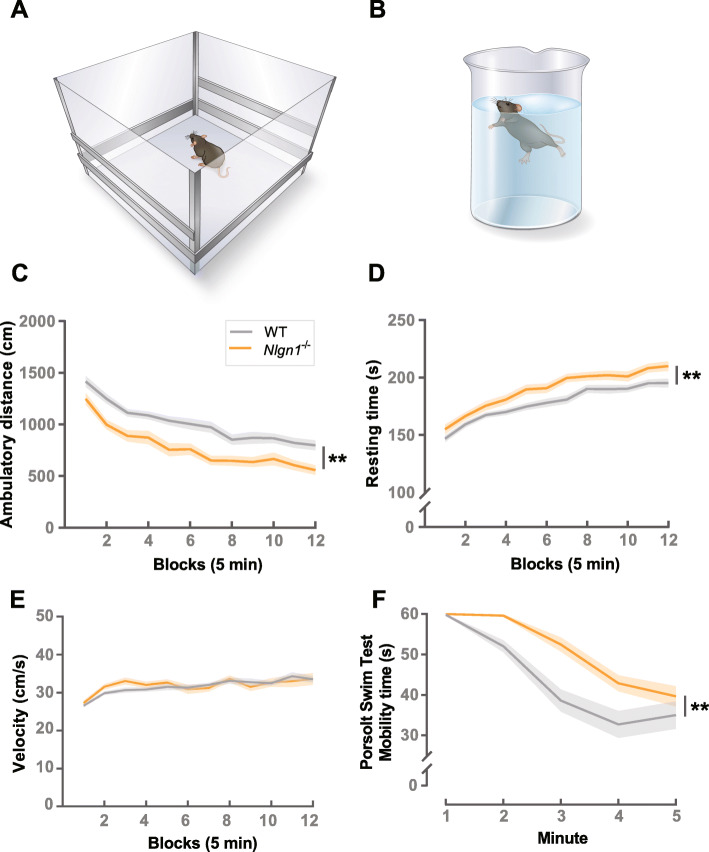
Measuring motivational behavior outside an operant environment. **a** Open-field test. **b** Porsolt swim test. *Nlgn1*^−/−^ mice (with previous operant experience) show *decreased* exploration and spontaneous locomotor activity in a novel, open-field environment. **c** Ambulatory distance (centimeters) (generalized linear model **p < 0.005) and **d** resting time (seconds) (mixed-effects linear model **p < 0.005) but no changes in **e** velocity (centimeters/second) (mixed-effects linear model). Values represent means ± SEM. **f** *Nlgn1*^−/−^ mice showed *increased* mobility time (seconds) in the Porsolt swim test; two-way ANOVA, time bins collapsed for analysis, main effect of genotype **p < 0.005, values represent means ± SEM

To investigate the generalizability of the observed motivational phenotype, we wanted to next examine whether *Nlgn1*^−/−^ mice were also less willing to exert effort outside an operant environment. We assessed exploration and spontaneous locomotor activity in a novel, open-field environment (Fig. 5a) (in darkness) to measure the decision between exploration and resting. We tested two separate cohorts of animals, one group that had previously been tested in an operant paradigm and a second experimentally naive group. Interestingly, in the group that had previous experimental experience, we see that *Nlgn1*^−/−^ mice travel a shorter distance (Fig. 5c) and spent more time resting (Fig. 5d) compared to WT controls, consistent with a reduced willingness to overcome the cost of physical effort. We noted that when we assessed the naive cohort of animals, we did not see a significant effect of genotype (Additional file 1: Fig. S14A-B) suggesting these measures of exploratory behavior are strongly influenced by previous experience. However, in both groups, movement velocity of *Nlgn1*^−/−^ mice in the open-field arena was either not different (Fig. 5e) or greater than WT controls (Additional file 1: Fig. S14C). This was further supported by no differences in the latencies to fall off an accelerating rotarod across repeated trials (Additional file 1: Fig. S15) highlighting loss of Nlgn1 does not impair intrinsic motor function; therefore, the observed changes in motivational measures are not due to locomotor capacity.

Motivation often refers to the ability to overcome physical effort to achieve a desirable outcome [46–49], but what if the desired outcome was to avoid punishment? We were therefore interested to know whether an increased aversion to physical effort to earn rewards would manifest as a decreased willingness to avoid punishment. To examine this, we next employed the Porsolt forced swim test (Fig. 5b) in which the choice to swim or “struggle” to escape the aversive situation of being immersed in water compared to immobility is taken to model behavioral despair [50–52]. Here, we surprisingly observed that *Nlgn1*^−/−^ mice spent significantly *more* time mobile than WT controls (Fig. 5f). These data suggest that the behavioral phenotype of *Nlgn1*^−/−^ mice cannot be described simply as a general reduction in the willingness to overcome effort cost.

### Convergence on a model of increased weighting on negative utilities

Theoretical accounts for the potential cognitive mechanisms underlying decreased motivation have been reported [44, 53, 54]. We therefore considered the possibility that the seemingly opposing motivational phenotypes observed in reward- and punishment-driven contexts many converge on the same underlying mechanism. On reflection, in the Porsolt swim test both the fear of drowning and the effort of swimming incurs negative utilities. Therefore, Nlgn1 may not only be involved in estimating the cost of physical effort, but rather more broadly important for regulating the sensitivity to domain-general negative utilities (any undesirable consequences of actions/inaction). To explore this, we sought a theoretical model to capture the key behavioral observations of *Nlgn1*^−/−^ mice across tasks including (1) normal binary effort-matched choices between a correct and incorrect response in learning tasks (e.g., visual discrimination, object-location paired associate, reversal), (2) fewer responses emitted on a sequential fixed ratio task (e.g., FR5–40), and (3) higher mobility when immersed in water in the Porsolt swim test. We visualized the behavioral predictions of the theory by simulating the behavior of a simple reinforcement learning model using the three tasks mentioned, allowing us to describe precisely and unambiguously the assumptions, architecture, and predictions of the theory. We compared the simulated effect of changing the parameters in the theoretical model to the effect of Nlgn1 deletion in the experimental data.

**Fig. 6:**
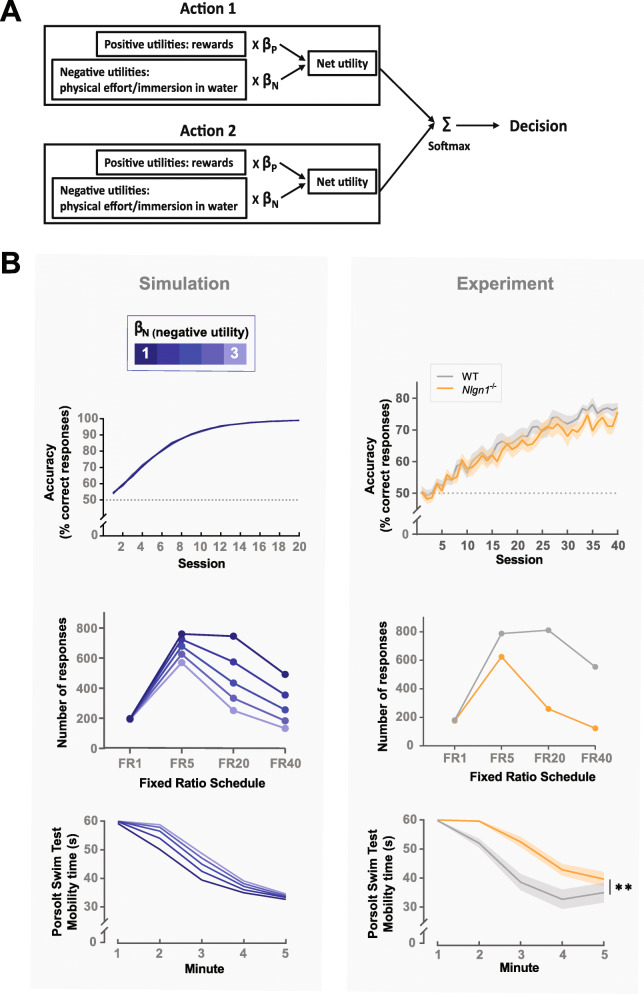
A model of increased weighting on negative utilities captures the observed *Nlgn1*^−/−^ behavioral phenotype across tasks. **a** An agent selects actions by comparing the net utility of each available action, sum of positive and negative utilities weighted by two separate parameters (*β*_P_ and *β*_N_). The model assumes different types of positive and negative utilities are under the general control of *β*_P_ and *β*_N_ (e.g., *β*_N_ affects both the weighting on physical effort as well as immersion in water where appropriate). Net utilities of potential actions are then fed into a softmax function for probabilistic action selection where actions with higher net utilities are selected with higher probabilities. **b** Left panel: Increasing *β*_N_ has (i) no impact on the choice between correct and incorrect responding in the simulated binary choice task, (ii) reduces the number of responses made in the simulated fixed ratio task where the choice is between responding (high-effort action) and resting (low-effort action), and (iii) increases mobility in the simulated Porsolt swim test where the choice is between swimming (high-effort action) and resting (low-effort action). **b** Right panel: For comparison, our experimental data from (i) object-location paired associate learning (PAL) task, (ii) fixed ratio task, and (iii) Porsolt swim test

In the model, the simulated agent selects actions by comparing the net utility of each available action, sum of positive and negative utilities weighted by two separate parameters (inspired by Collins and Frank [45], Fig. 6a). Importantly, these parameters affect only the weightings on learned action utilities but not the process of learning itself, allowing a potential dissociation between learning and action selection. We first considered how reducing the weighting on positive utilities (*β*_P_) would impact the calculation of net utilities (Additional file 1: Fig. S16). Reducing the weighting on positive utilities promotes low-effort-low-reward actions (e.g., resting) leading to a reduction in responses made in the fixed ratio task, consistent with our observed experimental data in *Nlgn1*^−/−^ mice. On the contrary, reducing the weighting on positive utilities renders the choice between correct and incorrect responding more random in the simulated binary choice task, because performance accuracy depends on the difference between the positive utilities associated with the correct and incorrect response. Here, we see this model does not capture the *Nlgn1*^−/−^ phenotype in the pairwise visual discrimination, object-location paired associate learning, and reversal learning tasks.

Next, we considered how increasing the weighting on negative utilities (*β*_N_) impacts calculation of net utilities (Fig. 6b). We found increasing *β*_N_ (i) did not alter the learning curves for binary choices because correct and incorrect responding required the same physical effort, (ii) reduced the number of responses in the simulated fixed ratio tasks because the potential rewards of responding were more heavily discounted by the physical effort incurred, and (iii) increased mobility in the Porsolt swim test because increasing *β*_N_ had a greater effect on greater punishment, under the assumption that immersion in water has a greater negative utility than the physical effort of swimming. Together, an increased weighting on negative utilities can, at least in principle, capture the divergent phenotypes across reward- and punishment-driven tasks to suggest loss of Nlgn1 may be important for balancing the weighting on positive and negative utilities in reward-cost trade-off.

## Discussion

Building on the extensive work on the molecular and signaling functions of Nlgn1 at the synapse, we investigated how the loss of Nlgn1 might impact components of decision-making. We found that Nlgn1 was not required for learning complex associative structures, or the subsequent updating of learned associations. However, *Nlgn1*^−/−^ mice were consistently *less* motivated to overcome the effort cost to earn rewards across different reward-based free-operant tasks, but *more* motivated to exert effort to avoid an inescapable aversive situation. We suggest these divergent phenotypes converge on a model of increased weighting on negative utilities, highlighting a novel valence-dependent role of Nlgn1 in reward-cost trade-off. Our findings provide novel evidence that updates current views to show Nlgn1 is essential for regulating distinct cognitive processes underlying decision-making. This work also demonstrates that learning and motivational processes can be dissociated.

It is widely held that NMDA receptor function and long-lasting forms of synaptic plasticity are required for various forms of learning (e.g., [28–31, 35, 55]). Based on previous findings that loss of Nlgn1 robustly impairs NMDA receptor function and synaptic plasticity in various brain regions (e.g., [7, 14, 15, 17–20, 25, 26]) and decreases spatial learning and memory in the Morris water maze [14], we initially speculated that Nlgn1 loss of function would likely impair the ability to learn complex associations in our various touchscreen-based tasks. Indeed, disrupting NMDA receptor signaling and plasticity has been shown to impair performance in these touchscreen-based tasks employing training parameters similar to our study [35, 55–58]. It is therefore striking that in our studies *Nlgn1*^−/−^ mice showed normal acquisition of complex associative structures and were able to modify learned associations. Towards collectively reconciling these findings, our data emphasize the capacity for synaptic signaling and plasticity does not map uniformly onto all forms of learning, thus highlighting the complexity in reducing behavioral processes to discrete cellular mechanisms. In line with this, Nlgn1 overexpression enhances NMDA receptor transmission without impairing LTP but nonetheless decreases learning and memory in the Morris water maze [24]. Given the many different levels of neural architecture between behavior and synaptic molecules, as well as the multiple components and interactions within each level, our findings caution interpretations between behavior and synaptic signaling and emphasize the relationship between the cellular mechanisms and the emergence of distinct forms of cognitive behavior is highly complex and indirect.

It is also worth noting that functional studies on synaptic proteins (including Nlgn1) have predominantly been performed in vitro and often limited to isolated brain regions (e.g., the hippocampus) and cell types (e.g., pyramidal neurons). Nlgn1 is predominantly expressed at excitatory synapses, but it has also been documented in oligodendrocytes [59] and astrocytes [60] and potentially plays a role in inhibitory synapse formation [61]; thus, unraveling cell-type-specific roles of neuroligins presents another layer of complexity (e.g., [62, 63]). The synaptic mechanisms required for the emergence of large-scale neural representations during complex learning, and how these might be regulated by molecular components that structurally organize synapses therefore remains largely unknown. One way forward would be to examine network-level synaptic changes during behavior. Technological advances in recent years now allow in vivo monitoring of neural dynamics of large populations of cells across multiple brain regions in awake-behaving animals with high cellular specificity. These emerging approaches offer new opportunities for probing how synaptic mechanisms influence large-scale neural dynamics underlying complex behavior.

Despite an intact capacity to acquire and flexibly update learned information, we found that loss of Nlgn1 alters motivational processing, impacting reward-cost trade-off. Action selection generally requires weighing up both the expected positive and negative consequences of available actions. We see a *reduced* willingness to overcome response effort for reward in touchscreen-based tasks. Theoretical models have been proposed to address the dissociation between learning and motivation for rewards, potentially explaining our dissociation [44, 54]. According to these theories, an animal could be less motivated to overcome the effort cost of responding yet maintain the normal ability to choose between correct and incorrect responses due to either an underestimation of the average reward rate of the environment independent of any particular action, or an overestimation of the effort costs of potential actions. However, we additionally see an *increased* willingness to exert effort to escape an aversive situation in the Porsolt swim test. One interpretation may be that this is due to an exaggerated anxiety or fear response. However, there is no strong evidence to suggest that loss of Nlgn1 elevates response to aversive stimuli. Previous work has shown loss of Nlgn1 does not alter freezing behavior during contextual and cued fear conditioning [14], though knockdown of Nlgn1 in the amygdala of rats impairs the retention of fear memory [19]. *Nlgn1*^−/−^ mice also display normal behavior on various anxiety-related assays [14].

The divergent phenotypes observed in the reward-associated touchscreen tasks and the punishment-associated Porsolt forced swim test could be due to either changes in distinct or shared underlying mechanisms involved in decision-making processes. Our behavioral simulation data show that the seemingly opposing phenotypes could, in principle, converge on an increased weighting on negative utilities. Two key questions arise from this interpretation. First, is it plausible to assume a common currency for effort cost and other forms of punishment? Conceptually, this seems reasonable since physical cost, like other forms of punishment, is a decision variable which animals should seek to minimize all else being equal [64]. Empirically, functional magnetic resonance imaging (fMRI) data suggest that hemodynamic responses in select brain regions such as the anterior insula cortex correlate with both physical cost and other forms of punishment [64–68]. Second, is it plausible that the brain affords dissociable neural computations and hardware for reward and punishment? It is generally accepted that there is at least partial dissociation between the neural implementation of reward and punishment [69–71]. Notwithstanding the lack of a consensus, reward and punishment processing has been shown to be differentially implemented by activity [72] and identity of distinct subsets of dopaminergic neurons [73–75] or opponent dopaminergic and serotoninergic signaling [54, 76].

We acknowledge that our proposed model of increased weighting on negative utilities is, at this point, tentative and requires further investigations. Indeed, our intention was to additionally assess whether *Nlgn1*^−/−^ mice would exert more effort to avoid punishment in an operant paradigm comparable to our touchscreen tasks. To address this, we attempted to train mice to press a lever to either avoid an upcoming footshock or to terminate it. Unfortunately, C57BL/6 mice were unable to acquire the action-safety contingency in both task variants. It appears that in the face of an aversive outcome, mice can escape but struggle to acquire trained actions to prevent it. This phenotype was also recently reported in rats [77] showing that while rats froze in response to footshock and the conditioned cue, they did not robustly detect the contingency between lever-press and footshock. Future studies on positive/negative utility trade-off will benefit from the development of robust aversive instrumental paradigms for rats and mice.

Behavior is complex; thus, it is not surprising that a single behavioral assay cannot often reliably assess the cognitive construct in question. Our data, cultivated from detailed analyses across a battery of tests, highlight the value of a deep behavioral dissection that enables the synthesis of a cross-task interpretation from multiple paradigms. These touchscreen tests provide a unique opportunity to obtain such a comprehensive dataset consisting of multiple behavioral measures that are unique and shared across tests, assessed within the same testing environment in a controlled and comparable manner. Extending previous work using these touchscreen operant paradigms, we also modified our data analysis approach to employ trial-by-trial level analyses with regression models. This approach is tailored to describing complex behavioral data sampled at the trial level and across multiple sessions, yet it is under-utilized in rodent behavioral studies where session or stage-level summary measures are commonly calculated, potentially missing valuable depth in data. Exploiting this trial-level approach, we incorporated various latencies into the analysis of free-operant behavior which more accurately represents the paradigm as a continuous decision process rather than discrete trials. We also show, for the first time, that we can further dissect response latencies to sub-components that differentially contribute to decision-making and learning (e.g., stimulus-selection vs stimulus-approach latency). Measuring latencies for different response epochs during behavioral responding within the touchscreen chambers is a powerful parameter, analogous to human cognitive measures such as processing speed and reaction times, which have been difficult to be capture in other rodent behavioral assays.

Finally, human mutations in *NLGN* genes, including *NLGN1*, have been reported in neurodevelopmental disorders including autism spectrum disorder [78, 79]. It is noteworthy that depression, affective, and anxiety symptoms are highly comorbid with neurodevelopmental disorders, supporting the need to dissect distinct components of cognitive behavior and gain a deeper understanding of the transdiagnostic psychological processes that can be effectively used as behavioral markers of disease.

## Conclusions

Our work updates canonical views of Nlgn1, a key postsynaptic cell-adhesion molecule, in cognitive behavior to show its critical for tuning valence-dependent processes regulating motivation, but not learning and updating complex associative structures. Our observations emphasize the relationship between the cellular mechanisms that support the emergence of distinct forms of cognitive behavior is highly complex and indirect; thus, the need to scrutinize established interpretations that impaired synaptic transmission/plasticity necessarily and uniquely lead to learning deficits. We highlight the value and advantage of our detailed behavioral dissection, exploiting a battery of free-operant touchscreen-based tests that enables the synthesis of a cohesive behavioral interpretation by identifying cross-task phenotypes. Extending this, we showcase response latencies can be dissected to examine their differential contribution to decision-making and learning, providing avenues for capturing different response epochs during behavioral responding analogous to human cognitive measures such as processing speed and reaction times that are not accessible in most rodent behavioral assays. We hope our analyses and approaches provide useful tools as the neuroscience community expand the integration of in vivo recordings and imaging during complex cognitive behavior in systems neuroscience. We demonstrate that learning and motivational processes can be dissociated in an animal model, providing insights into how human mutations in synapse genes that are expressed throughout the brain can selectively impact specific cognitive constructs, thus manifesting as disruptions in distinct symptoms. Of importance, depression, affective, and anxiety symptoms are highly comorbid with neurodevelopmental disorders. Our work provides evidence of being able to dissect distinct components of cognitive behavior in preclinical animal models, towards having robust models that enable deeper understandings into transdiagnostic behavioral markers of disease.

## Methods

### Animals and housing

Heterozygous *Nlgn1*^+/−^ mice were obtained from Prof. Nils Brose, generated by homologous recombination of embryonic stem cells deleting exon sequences covering the translational start site and 546 bp of 5′ coding sequence of the murine *Nlgn1* gene [80], and backcrossed more than 10 generations on a C57BL/6 background. *Nlgn1*^−/−^ mice and WT littermate matched controls were generated at The Florey by mating heterozygous females and males. Mice were weaned at 3–4 weeks of age and housed in mixed genotype groups of 2–4 per cage with food and water available ad libitum. Bedding consisted of sawdust chips 2 cm deep and tissue paper for nesting material. At ~ 10 weeks of age, mice were moved from individually ventilated cages to open-top cages in a humidity and temperature-controlled holding room maintained on a 12:12-h reversed light/dark cycle (lights off at 07:00). Mice were acclimatized to these conditions for a minimum of 1 week prior to handing. Pre-training began at ~ 12 weeks of age. All behavioral testing was conducted during the dark active phase of the cycle, with the experimenter blinded to genotype during behavioral testing. All procedures were approved by The Florey Institute of Neuroscience and Mental Health Animal Ethics Committee.

#### Cohorts of mice used for behavioral testing

A total of 6 cohorts of mice were used in the present study (see Additional file 1: Fig. S1 for a schematic of sequence of tasks for each cohort). Cohort 1 (WT: *n* = 12 female/*n* = 15 male; *Nlgn1*^−/−^: *n* = 13 female/*n* = 13 male) was tested in the pairwise visual discrimination, reversal learning, object-location paired associate learning, and extinction learning tasks. When a single cohort of animals was tested on multiple touchscreen-based tasks, mice were placed back on free-feeding for ~ 2 weeks and baseline weights updated prior to commencing food restriction for the next task. Cohort 2 (WT: *n* = 14 female/*n* = 14 male; *Nlgn1*^−/−^: *n* = 14 female/*n* = 17 male) was tested in the fixed ratio task (FR1–40) with strawberry milk rewards and fixed ratio 20 (FR20) task with water rewards. Cohort 3 (WT: *n* = 11 female/*n* = 12 male; *Nlgn1*^−/−^: *n* = 7 female/*n* = 12 male) and cohort 4 (WT: *n* = 6 female/*n* = 4 male; *Nlgn1*^−/−^: *n* = 6 female/*n* = 4 male) were tested in the progressive ratio task, spontaneous locomotor activity, and accelerating rotarod tests. Cohort 5 (WT: *n* = 13 female/*n* = 10 male; *Nlgn1*^−/−^: *n* = 10 female/*n* = 12 male) was tested in the Porsolt forced swim test following ~ 2 weeks simple operant training for a different study not included in this paper. Cohort 6 (WT: *n* = 13 female/*n* = 16 male; *Nlgn1*^−/−^: *n* = 11 female/*n* = 16 male) was experimentally naive and tested for spontaneous locomotor activity. For all non-touchscreen-based tasks, mice were not food restricted when tested.

### Rodent touchscreen operant tasks

#### Apparatus

Touchscreen testing was conducted in the Bussey-Saksida mouse touchscreen operant system (Campden Instruments Ltd., UK). Stimulus presentation, task parameters, and data recording were controlled through Whisker Server and ABET II Touch software (Campden Instruments Ltd., UK). The two-hole mask was used for the pairwise visual discrimination and reversal learning tasks, and the three-whole mask used for the object-location paired associate learning, extinction learning, fixed ratio, and progressive ratio tasks.

#### Touchscreen pre-training

Pre-training and food restriction were conducted as previously described [36, 37, 42]. Before testing, mice were first food restricted to 85–90% free-feeding body weight. Mice were then trained through five phases for instrumental conditioning to learn to selectively nose-poke stimuli displayed on the touchscreen in order to obtain a liquid reward (strawberry milk, Devondale, Australia; 20 μl rewards for all touchscreen tests). All animals received one daily session for all touchscreen testing. Mice were required to reach a set performance criterion for each phase before advancing to the next phase. Briefly, mice were habituated (phase 1, Habituation) to the touchscreen chamber and to consuming liquid rewards from the reward magazine or receptacle for two 30-min sessions (criterion = consume 200 μl of liquid reward freely available in the reward receptacle at each session). For phases 2–5, a trial did not advance until the reward was consumed. In phase 2 (Initial Touch) or the Pavlovian stage, a single visual stimulus was displayed on the screen for 30 s, after which the disappearance of the stimulus coincided with delivery of a reward (20 μl), presentation of a tone and illumination of the reward receptacle (criterion = 30 trials within 60 min). A nose-poke response to the stimulus during the 30-s window was rewarded with 3 times the reward amount to encourage responding. In phase 3 (Must Touch), mice had to nose-poke visual stimuli displayed on the screen to obtain a reward (criterion = 30 trials within 60 min). Mice then learned to initiate a new trial with a head entry into the reward receptacle (phase 4, Must Initiate, criterion = 30 trials within 60 min). In phase 5, responses at a blank part of the screen during stimulus presentation produced a 5-s timeout (signaled by illumination of the house light and no delivery of reward) to discourage indiscriminate responding (criterion = 21/30 correct responses within 60 min on 2 consecutive days). If another response to a blank part of the screen during stimulus presentation was made, there was a 5-s inter-trial interval (ITI), and then the same trial was repeated (the same stimulus presented in the same screen location, termed a “correction trial”) until the mouse made a correct response. Therefore, phases 2–5 consisted of 30 trials (pseudorandom first-presentation), and phase 5 also included an unlimited number of correction trials.

#### Pairwise visual discrimination and reversal learning

The pairwise visual discrimination (PD) and reversal learning (RL) tasks were conducted like that previously described [36, 37, 42]. Briefly, mice were trained to discriminate between two novel, equiluminescent visual stimuli (left and right diagonal stripes) displayed pseudorandomly across two locations with equal number of appearances at each location. Stimuli were 5 cm × 5 cm in size separated by 3 cm between stimuli and displayed 2 cm from the bottom of the touchscreen and ~ 5.5 cm away from the sides of the touchscreen. Response to one stimulus resulted in reward delivery (S+, correct response), followed by a pseudorandom trial (maximum 30 per session); response to the other stimulus resulted in a 5-s timeout, illumination of the house light followed by a correction trial. The same stimulus configuration was presented on correction trials until a correct response was made and a reward was delivered. Correction trials were not counted towards the trial limit or percentage of correct responses of a session. The designation of S+ and S− was counterbalanced within genotype and sex groups. Mice were trained to an acquisition criterion of ≥ 80% correct responses on two consecutive sessions. Following the acquisition of the visual discrimination task, mice were immediately moved on to the reversal leaning task, where the previously acquired reward contingencies were reversed. Reversal learning was assessed across 20 sessions.

#### Object-location paired associate learning

The object-location paired associate learning (PAL) task was conducted as previously described [36, 37]. Briefly, mice were trained to acquire reward associations jointly defined by visual stimuli (flower, plane, and spider) and their assigned correct spatial locations on the touchscreen (left, center, and right, respectively). Stimuli were 5 cm × 5 cm in size separated by 2 cm between stimuli and displayed 2 cm from the bottom and ~ 2.5 cm away from the sides of the touchscreen. For each trial, only two objects were presented: one object in its correct location (S+) and the other object in one of two incorrect locations (S−); therefore, there were six possible trial types. A nose-poke to the S+ resulted in delivery of a reward followed by a pseudorandom trial (maximum 36 per session), and incorrect responses resulted in a 5-s timeout followed by correction trial. Visuospatial learning in the PAL task was assessed across 40 sessions.

#### Instrumental extinction learning

The instrumental extinction learning task was conducted similar to that previously described [37, 42]. Mice were first trained to make a nose-poke response to a single white square displayed on the touchscreen (stimulus was 3 cm × 3 cm in size, displayed 3 cm from the bottom and ~ 10.5 cm away from the sides of the touchscreen) for a reward until reaching a set acquisition criterion (30 trials in < 12.5 min on five consecutive sessions). Following acquisition, instrumental extinction was assessed where responses were no longer rewarded (30 trials per session tested across 6 sessions). During extinction, the visual stimulus was displayed for 10 s on each trial and animals could either make a response or an omission.

#### Progressive ratio

Details on testing the touchscreen-based progressive ratio task have been described previously [81]. Briefly, mice had to make nose-poke responses to a single white square displayed on the touchscreen (stimulus was 4 cm × 4 cm in size, displayed 1.5 cm from the bottom and ~ 10 cm away from the sides of the touchscreen) for a reward. Naive mice first underwent phases 1 and 2 of touchscreen pre-training, followed by one session each of fixed ratio (FR) schedules of 1 (FR1), FR2, and FR3 and three sessions of FR5 training where a fixed number of nose-pokes (1, 2, 3, and 5 respectively) were required for a reward. Mice were required to complete 30 trials in 60 min in each of the FR sessions (criterion). Once training criterion was reached, mice advanced to the progressive ratio stage where the number of nose-poke responses required to obtain a reward incremented by 4 after every trial (1, 5, 9, 13, etc.,) until animals reach a breakpoint. If no responses to the touchscreen or entries to the reward receptacle were detected for 5 min, the session ended and the animal removed from the chamber. Mice were tested on 6 progressive ratio sessions.

#### Fixed ratios

Touchscreen-based fixed ratio testing was similar to that described for progressive ratio (mice had to make nose-poke responses to a single white square displayed on the touchscreen for a reward; stimulus was 4 cm × 4 cm in size, displayed 1.5 cm from the bottom and ~ 10 cm away from the sides of the touchscreen). Naive mice first underwent phases 1 and 2 of touchscreen pre-training followed by three sessions of FR1 and had to complete 30 trials within a 60-min session before advancing. During the next serial FR test stage, mice were given 60 min per session to make as many responses as they were willing to, and sessions did not terminate due to inactivity. Mice were tested on three sessions of FR1, FR5, FR20, and FR40 sequentially.

#### Fixed ratio with water rewards

Following the serial FR testing, mice were water-restricted with access to water limited to 1 h per day. Water-restricted body weights were maintained between 85 and 90% of free-feeding body weight. Mice were tested on a FR20 schedule where 20 nose-poke responses were required to deliver a water reward (20 μl) for three sessions. After each session, mice were returned to home cage and given 1-h free access to water.

#### Touchscreen latency measures

Across all our touchscreen tests, we assessed 4 latency measures (see Fig. 3a, Additional file 1: Fig. S4B). Initiation latency measures the time from the end of the inter-trial interval to trial initiation by head entry into the reward receptacle to commence a trial. Head entry triggers the presentation of stimuli. Stimulus-approach latency measures the time from exiting the reward receptacle to arriving in front of the touchscreen (breaking the front IR beam). Stimulus-selection latency measures the time from arriving in front of the touchscreen to nose-poking one of the stimuli on the touchscreen. Lastly, reward collection latency measures the time from delivery of the reward tone to head entry into the reward receptacle.

### Non-operant behavioral tests

#### Spontaneous locomotor activity

Mice were assessed for spontaneous locomotor activity in a novel open-field arena (27.31 cm (L) × 27.31 cm (W) × 20.32 cm (H), Med Associates, St. Albans, VT, USA) using the Activity Monitor system and software (Med Associates, St. Albans, VT, USA). Animals were tested in darkness (to promote exploration) for 60 min to provide an adequate time window to capture the habituation of locomotor activity to a plateau level.

#### Accelerating rotarod

For motor coordination and learning on the accelerating rotarod, mice were exposed to three 5-min trials across 3 consecutive days (9 trials in total). Mice were placed on a rotating rod (Ugo Basile, Gemonio, VA, Italy) facing forward (against the rotating direction of the rod) before acceleration started. Subsequently, the speed of the rotating rod accelerated from 4 to 40 rpm and latency to fall off was manually recorded. Falls before the acceleration started were not recorded as failures. Passively rotating by clinging onto the rod was recorded as falls. Testing was conducted under low lighting settings (20 lx red light).

#### Porsolt forced swim test

Mice were individually placed into a beaker (13 cm diameter) with 1.6 L of water (23–25 °C) for a single 5-min session under ambient lighting (20–25 lx white light). Each session was video-recorded, and total mobility time throughout the 5-min session was measured (no time bins excluded). Scoring was obtained using the automated ForcedSwimScan software (CleverSys Inc., VA, USA) under previously optimized settings [82] eliminating the need for manual observer scoring.

### Data analysis

Multi-session touchscreen choice data were analyzed with generalized (logistic) linear mixed models. This is motivated by (1) trial-by-trial binary nature of the data, (2) the need to estimate learning rates per time unit (session/trial), and (3) the non-linearity of learning curves. Touchscreen latency data across sessions were analyzed with quantile regressions to assess distribution-wide differences.

Effect size of task variables (stimulus location, session, correction trial, etc.), biological variables (genotype and sex), and interactions between a subset of variables (genotype × sex, genotype × session, etc.) on behavioral measures (accuracy, latencies, etc.) were estimated together with 95% confidence intervals (CI) and statistical significance using various two-level mixed-effect general, generalized linear models or quantile regression (StataCorp, TX, USA). Mice were treated as level 2 clusters and random intercepts. Binary performance measures (correct/incorrect response, response/omission) were analyzed trial-by-trial using the generalized linear latent and mixed models (GLLAMM) program [38] with a logit link function, whereby the effects of task variables were expressed as odds ratios with an odds ratio of 1 indicating no effect (e.g., an effect of session > 1 indicates response accuracy improves over sessions). Latency data were analyzed using quantile regressions with robust and clustered standard errors [83] from the 0.05 to 0.95 quantile at 0.05 steps to allow distribution-wide comparisons (see Additional file 1: Fig. S6), whereby effects of task variables were expressed as latency difference with 0 indicating no effect (e.g., an effect of genotype > 0 for a given quantile indicates *Nlgn1*^−/−^ mice have longer latencies).

For spontaneous locomotor activity, ambulatory distance was analyzed with GLLAMM with a log link. Other performance measures were analyzed using a mixed-effects linear model if the performance measures were normally distributed or median regressions otherwise [83].

To analyze the effect of correction trials and reoccurring pseudorandom trials on accuracy, two additional binary variables were included in the models indicating whether a trial is a correction trial/reoccurring trial (correction trials were excluded in estimating the effect of reoccurring pseudorandom trials). Heteroskedasticity-robust standard errors adjusted for clustering within animals were used for all analyses.

### Behavior simulation

#### Agent

A simple reinforcement learning agent learned the utility of an action following the classic Rescorla-Wagner rule [84]:

1 $$ {Q}_{t+1}(A)={Q}_t(A)+\alpha \bullet \left[{r}_t-{Q}_t(A)\right] $$

Here, *Q*_t_(*A*) is the learned utility of a given action *A* on trial *t*, *α* is the learning rate, and *r*_*t*_ is the reinforcement received on trial *t*. Actions can have both positive and negative utilities (e.g., responding may result in rewards but also incurs effort). The net utility of a given action is given by the linear combination of its positive and negative utilities, the relative importance of which is controlled independently by *β*_*P*_ and *β*_*N*_ respectively:

2 $$ U(A)={\sum}_i\left[I\bullet {\beta}_P+\left(1-I\right)\bullet {\beta}_N\right]\bullet {Q}_i(A) $$

Here, *U*(*A*) is the net utility of action *A*, *Q*_*i*_(*A*) is the different positive or negative utilities of *A*, *I* is the indicator function:

3 $$ I={\mathbbm{1}}_{Q\left({A}_i\right)\ge 0}\left(Q\left({A}_i\right)\right) $$

Such that,

$$ I=\left\{\begin{array}{c}1\  if\ Q\left({A}_i\right)\ge 0\ \\ {}0\  if\ Q\left({A}_i\right)<0\end{array}\right. $$

Action selection is given by a softmax function on the net utilities of potential actions

4 $$ P(A)=\frac{e^{U(A)}}{\sum \limits_i{e}^{U\left({A}_i\right)}} $$

Here, *P*(*A*) is the probability of choosing action *A*, which depends on the net utility of *A* compared to that of alternative actions. For all our simulations, a choice is made between only two actions.

#### Simulations

##### Binary choice (two-armed bandit) task

The reinforcement learning agent learned to choose between a correct and an incorrect response for 30 trials per session across 20 sessions. The correct response was always rewarded, and the incorrect response never rewarded. Both correct and incorrect responding incurred a negative utility of − 1 representing the physical effort of responding.

##### Serial fixed ratio task

The agent was trained sequentially through FR1, 5, 20, and 40 for three sessions on each ratio requirement where it chose between responding or resting. Responding resulted in a reward if the ratio requirement was met (positive utility) and incurred a negative utility of − 1 representing the physical effort. Resting results in no reward but incurs a much smaller effort-related negative utility of − 0.2. Note that the designation of the alternative action as resting is arbitrary. The general idea is that an animal chooses between responding and other low-reward-low-effort actions. Time elapsed as the agent chose to either respond or rest, the session ended after 2700 timesteps roughly corresponding to a 2700-s or 45-min session.

##### Porsolt swim test

The forced swim test was simulated as a choice between swimming and resting. Swimming was initialized with a utility of 0 representing that the agent initially believed that swimming will lead to a neutral outcome, and a utility of − 1 represents the effort of swimming. Resting had a large negative utility of − 10 representing the possibility of drowning but incurred no effort. Every time the agent chose to swim, it received a reinforcement of − 9 thereby gradually learned by Eq. (1) that swimming did not markedly improve the situation therefore reduced mobility over time. For simplicity, the agent made 300 decisions over 300 timesteps roughly corresponding to a 5-min session.

Note the logic of the proposed model does not depend on the specific values of task parameters used for the simulations, which were chosen so that the behavioral simulations are quantitatively similar to the experimental data.

## Supplementary information

**Additional file 1: Fig. S1.** Sequence of tasks administered for different cohorts of *Nlgn1*^−/−^ and wildtype mice in this study. **Fig. S2.** *Nlgn1*^−/−^ and wildtype mice required similar number of sessions to acquire touchscreen pre-training. **Fig. S3.** Instrumental extinction learning curves. **Fig. S4.** Task dynamics and latency measures of the pairwise visual discrimination, reversal learning and object-location paired associate learning tasks. **Fig. S5.** Performance accuracy on trials across learning the pairwise visual discrimination, reversal learning and object-location paired associate learning tasks. **Fig. S6.** Latency data are highly skewed and poorly captured by a single summary measure. **Fig. S7.** Stimulus-selection latency positively predicts response accuracy but not stimulus-approach latency. **Fig. S8.** Latency learning curves. **Fig. S9.** Number of responses made in fixed ratio task. **Fig. S10.** Post-reinforcement pause and average response interval in fixed ratio task. **Fig. S11.** Response-by-response comparison of inter-response intervals between *Nlgn1*^−/−^ and wildtype mice. **Fig. S12.** Average number of responses to breakpoint in progressive ratio task. **Fig. S13.** *Nlgn1*^−/−^ mice show decreased number of responses for water rewards. **Fig. S14.** Experimentally naive *Nlgn1*^−/−^ mice show subtle changes in exploration and spontaneous locomotor activity in a novel, open-field environment. **Fig. S15.** *Nlgn1*^−/−^ mice displayed normal motor coordination and learning on the accelerating rotarod test. **Fig. S16.** Simulated effect of decreasing the weighting on positive utilities (β_P_) in the calculation of net utilities.

**Additional file 2: Table S1**. Variables included in regression models.

## Data Availability

Data that support the findings of this study have been made openly available using the Open Science Framework repository (https://osf.io/vfys8/, DOI: 10.17605/OSF.IO/VFYS8) and will also be available from MouseBytes (https://mousebytes.ca/home) [85]. Preprint of this manuscript was made available on bioRxiv on 02 January 2020 [86].

## Abbreviations

AMPA: α-Amino-3-hydroxyl-5-methyl-4-isoxazole-propionate
FR: Fixed ratio
GLLAMM: Generalized linear latent and mixed models
LTP: Long-term potentiation
Nlgn1: Neuroligin-1
*Nlgn1*^−/−^: Neuroligin-1 knockout
NMDA: *N*-Methyl-d-aspartate
PAL: Object-location paired associate learning
PD: Pairwise visual discrimination
PR: Progressive ratio
PSD: Postsynaptic density
RL: Reversal learning
WT: Wildtype
