# Supplementary material for: A molecular insight into the dissociable regulation of associative learning and motivation by the synaptic protein neuroligin-1
Source: BMC Biol. 2020 Sep 14;18:118. doi: 10.1186/s12915-020-00848-7 (PMC7646379; doi:10.1186/s12915-020-00848-7)
Supplement: Supplementary file 1 — Additional file 1: Fig. S1. Sequence of tasks administered for different cohorts of Nlgn1−/− and wildtype mice in this study. Fig. S2. Nlgn1−/− and wildtype mice required similar number of sessions to acquire touchscreen pre-training. Fig. S3. Instrumental extinction learning curves. Fig. S4. Task dynamics and latency measures of the pairwise visual discrimination, reversal learning and object-location paired associate learning tasks. Fig. S5. Performance accuracy on trials across learning the pairwise visual discrimination, reversal learning and object-location paired associate learning tasks. Fig. S6. Latency data are highly skewed and poorly captured by a single summary measure. Fig. S7. Stimulus-selection latency positively predicts response accuracy but not stimulus-approach latency. Fig. S8. Latency learning curves. Fig. S9. Number of responses made in fixed ratio task. Fig. S10. Post-reinforcement pause and average response interval in fixed ratio task. Fig. S11. Response-by-response comparison of inter-response intervals between Nlgn1−/− and wildtype mice. Fig. S12. Average number of responses to breakpoint in progressive ratio task. Fig. S13. Nlgn1−/− mice show decreased number of responses for water rewards. Fig. S14. Experimentally naive Nlgn1−/− mice show subtle changes in exploration and spontaneous locomotor activity in a novel, open-field environment. Fig. S15. Nlgn1−/− mice displayed normal motor coordination and learning on the accelerating rotarod test. Fig. S16. Simulated effect of decreasing the weighting on positive utilities (βP) in the calculation of net utilities. [file 12915_2020_848_MOESM1_ESM.pdf]

Additional File 1

A molecular insight into the dissociable regulation of associative learning and motivation by the synaptic protein neuroligin-1

Jiaqi Luo[1], Jessica M Tan[1], Jess Nithianantharajah[1]

1. Florey Institute of Neuroscience and Mental Health, Florey Department of Neuroscience, Melbourne Brain Centre, University of Melbourne, Parkville Victoria Australia.

Cohort 1	PD operant pre-training	Pairwise Visual Discrimination (PD)	Reversal Learning	Object-location Paired Associate Learning	Extinction Learning
Cohort 2	FR operant pre-training	Fixed Ratio (FR1-40) (strawberry milk reward)	Fixed Ratio (FR20) (water reward)		
Cohort 3 & 4	PR operant pre-training	Progressive Ratio (PR)	Spontaneous Locomotor Activity	Accelerating Rotarod	
Cohort 5	Operant training	Porsolt Forced Swim Test			
Cohort 6	Naive	Spontaneous Locomotor Activity			

Figure S1: Sequence of tasks administered for different cohorts of *Nlgn1*$^{-/-}$ and wildtype mice in this study. See Methods for details.

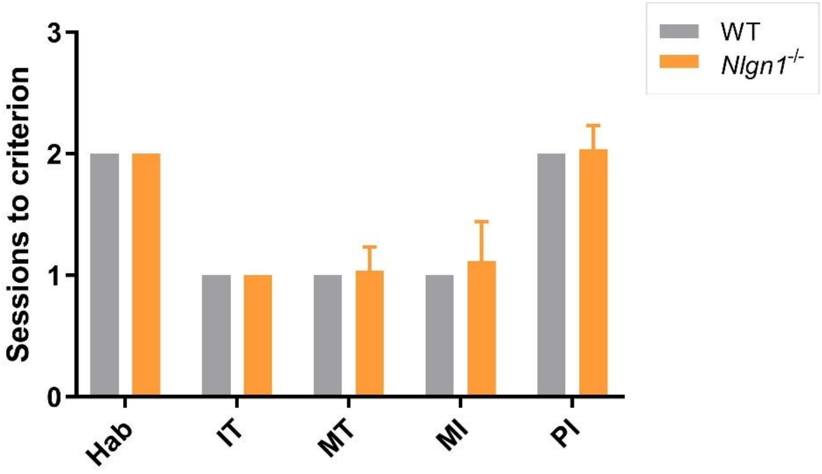

Figure S2: *Nlgn1*$^{-/-}$ and wildtype (WT) mice required similar number of sessions to acquire touchscreen pre-training. Mice were trained through five phases: (i) Habituation, Hab; ii) Initial touch, IT; iii) Must touch, MT; iv) Must initiate, MI; v) Punish Incorrect, PI to initiate trials and selectively nose-poke visual stimuli displayed on the touchscreen in order to obtain rewards (see Methods). A criterion for each phase had to be reached before advancing to the next phase.

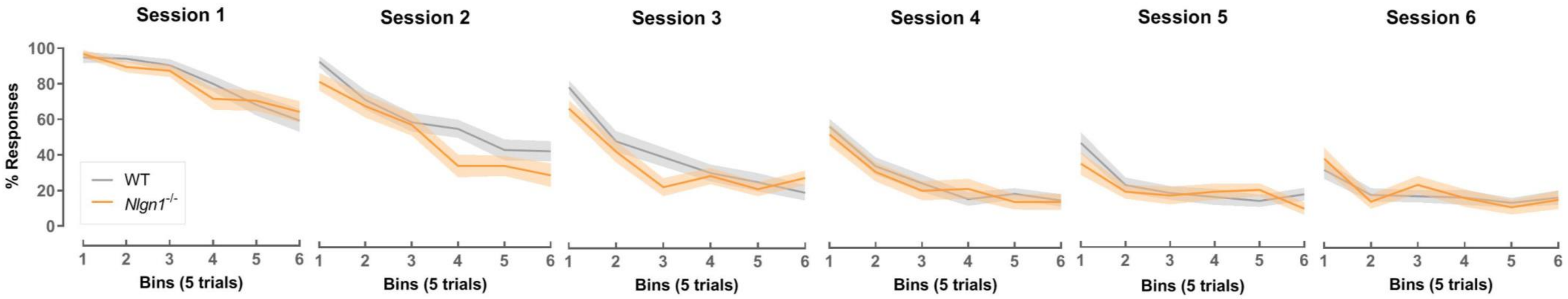

Figure S3: Instrumental extinction learning curves. Percentages of responses within a session (blocks of 5 trials) and across sessions in the instrumental extinction learning task. Values represent means ± SEM.

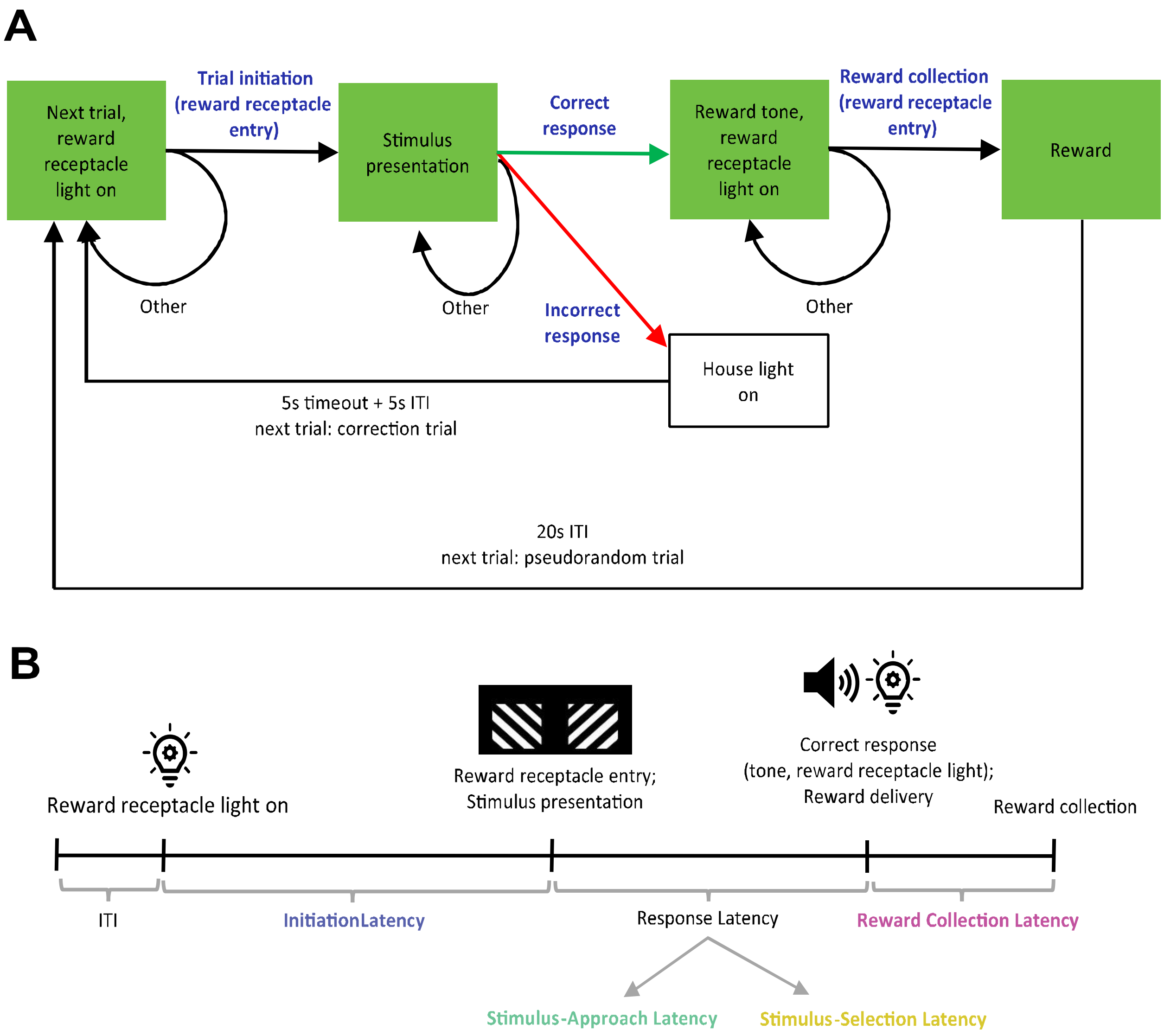

Figure S4: Task dynamics and latency measures of the pairwise visual discrimination (PD), reversal learning (RL) and object-location paired associate learning (PAL) tasks. (A) Task-state transitions. Green boxes represent task states and associated cues. Arrows represent transitions to the next state or staying in the current state. Blue text labels represent actions leading to transitions. ITI: inter-trial intervals. **(B)** Latency measurements. Illumination of the reward receptacle light signals the availability of the next trial after an ITI. Initiation latency measures the time from the end of ITI to trial initiation by head entry into the reward receptacle. Head entry triggers the presentation of stimuli. Stimulus-approach latency measures the time from exiting the reward receptacle to arriving in front of the touchscreen. Stimulus-selection latency measures the time from arriving in front of the touchscreen to nose-poking one of the stimuli. If the response is correct, a reward tone and the reward receptacle light signal the delivery of a reward. Reward collection latency measures the time from delivery of the reward tone to head entry into the reward receptacle.

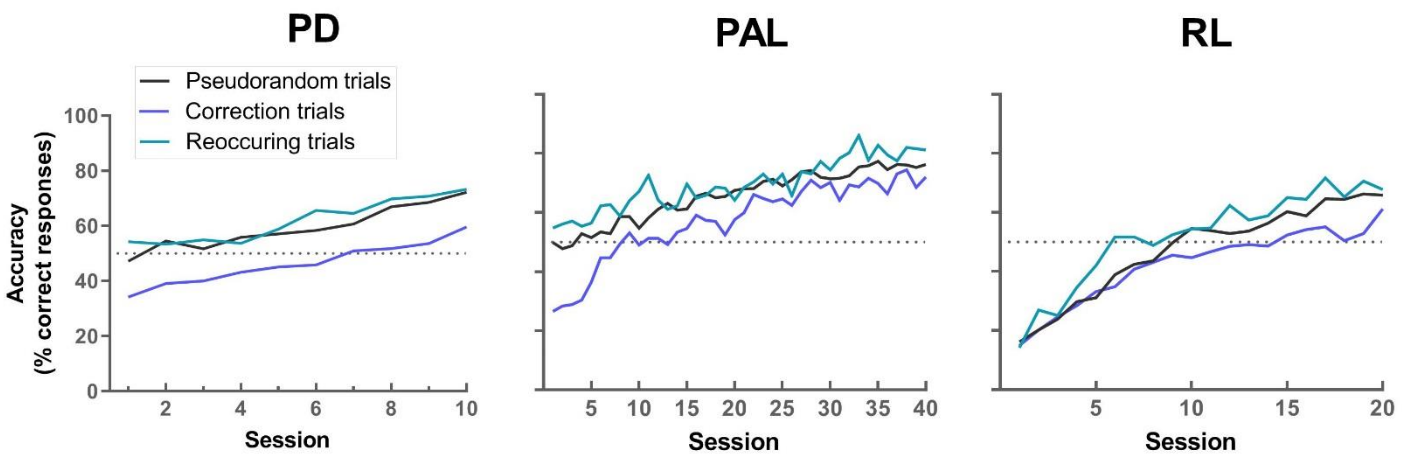

Figure S5: Performance accuracy on trials across learning the pairwise visual discrimination (PD), reversal learning (RL) and object-location paired associate learning (PAL) tasks. Population data showing accuracy of pseudorandom, correction and reoccurring trials across sessions on each task. A correct response to a first-presentation 'pseudorandom trial' was always followed by another pseudorandom trial, where the stimuli and location are displayed in a pseudorandom and counterbalanced manner. In contrast, an incorrect response was always followed by a 'correction trial' where the exact same stimulus-location configuration of that (pseudorandom) trial is repeatedly presented until mice switch their response to make a correct response and earn a reward. A 'reoccurring trial' is when the same stimulus-location configuration happened to reoccur on a consecutive trial. Mice were less accurate on correction trials suggesting a tendency to reselect the same incorrect response previously selected. Further, mice are more accurate on reoccurring trials showing they are more likely to reselect a correct response previously selected. Population data presented with genotype and sex combined as no genotype x sex interactions detected.

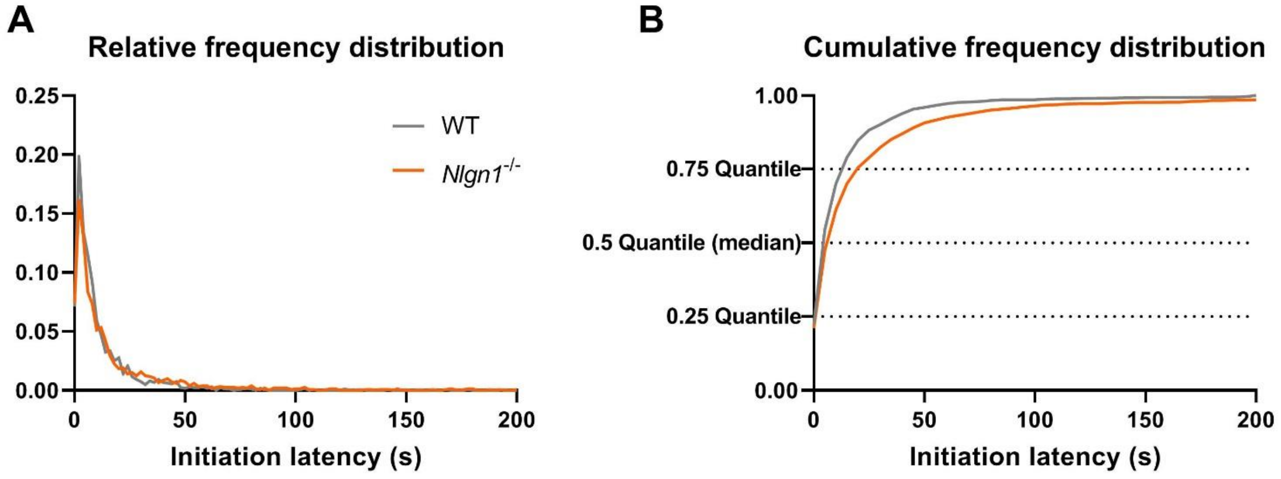

Figure S6: Latency data are highly skewed and poorly captured by a single summary measure. Distribution of initiation latency is presented as an example to show latency data are highly skewed and differences between experimental groups are not constant throughout the distribution. **(A)** Relative frequency distribution of latencies highlights using the mean as a summary measure for comparisons is inappropriate due to the long tails of the distributions. **(B)** Cumulative frequency distribution of latencies demonstrates the difference between groups (e.g., genotype) varies across different positions (quantiles) of the distributions (indicated by the size of the gap between the two curves at the same quantile).

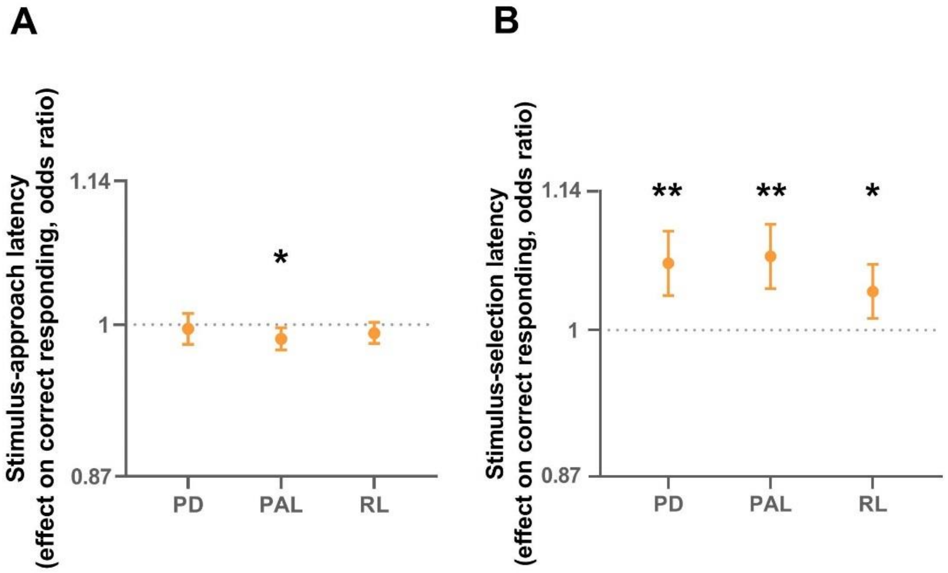

Figure S7: Stimulus-selection latency positively predicts response accuracy but not stimulus-approach latency. (A) Stimulus-approach latency does not positively predict response accuracy across pairwise visual discrimination (PD), object-location paired associate learning (PAL) and reversal learning (RL). However, **(B)** stimulus-selection latency positively predicted increased response accuracy across PD, PAL and RL. See Additional File 2, Table S1 for statistics. Logistic regression, *$p < 0.05$, **$p < 0.005$ significantly different from 1, values represent odds ratio ± 95% CI.

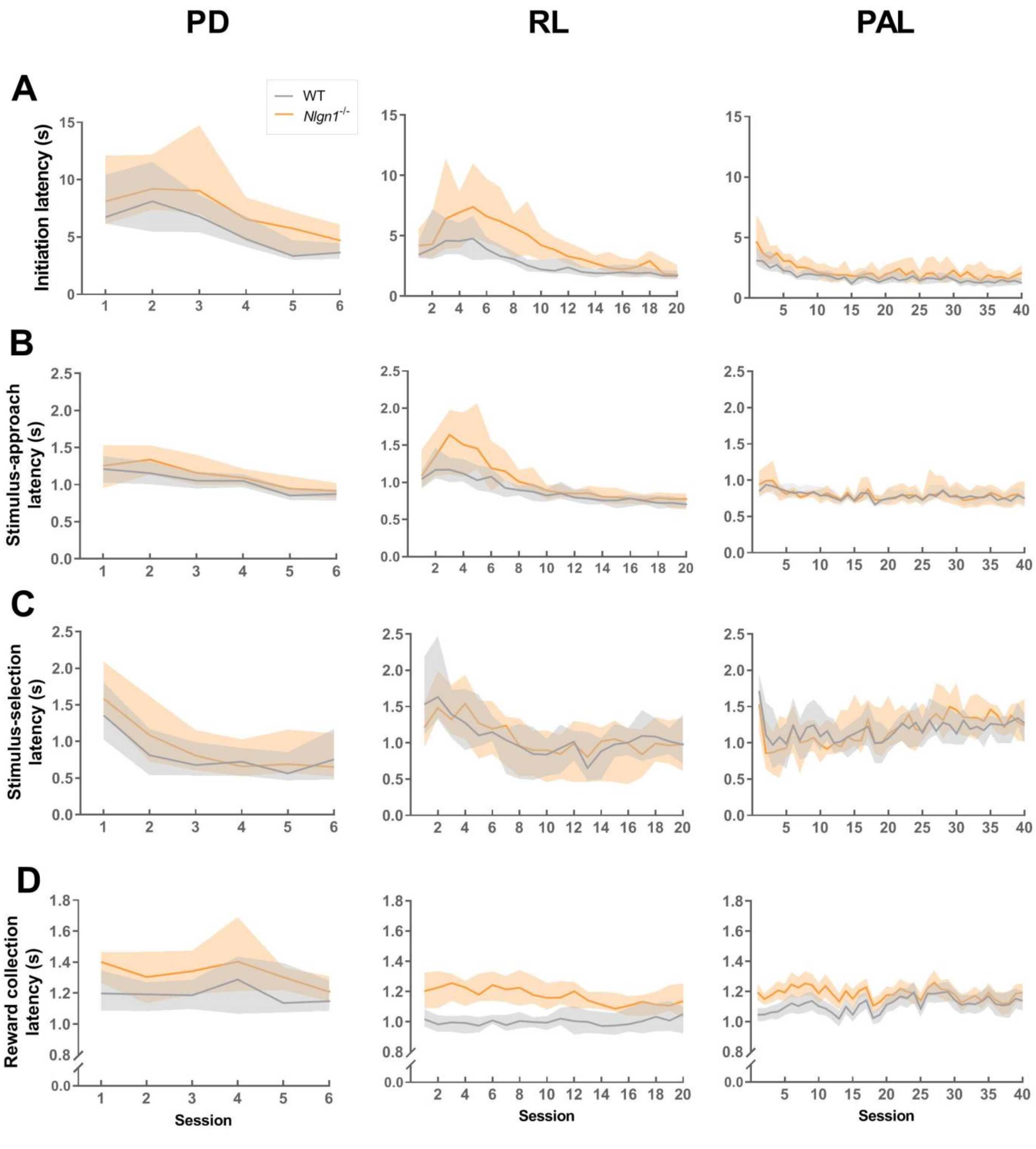

Figure S8: Latency learning curves. Learning curves of *Nlgn1*$^{-/-}$ and wildtype (WT) mice showing the median **(A)** initiation latency, **(B)** stimulus-approach latency, **(C)** stimulus-selection latency and **(D)** reward collection latency across pairwise visual discrimination (PD), reversal learning (RL) and object-location paired associate learning (PAL) tasks. Tasks arranged in columns (left to right) in order of training. Only the first 6 sessions of PD containing all mice visualised (prior to some mice subsequently advancing after reaching criterion). Values represents median ± 95% CI.

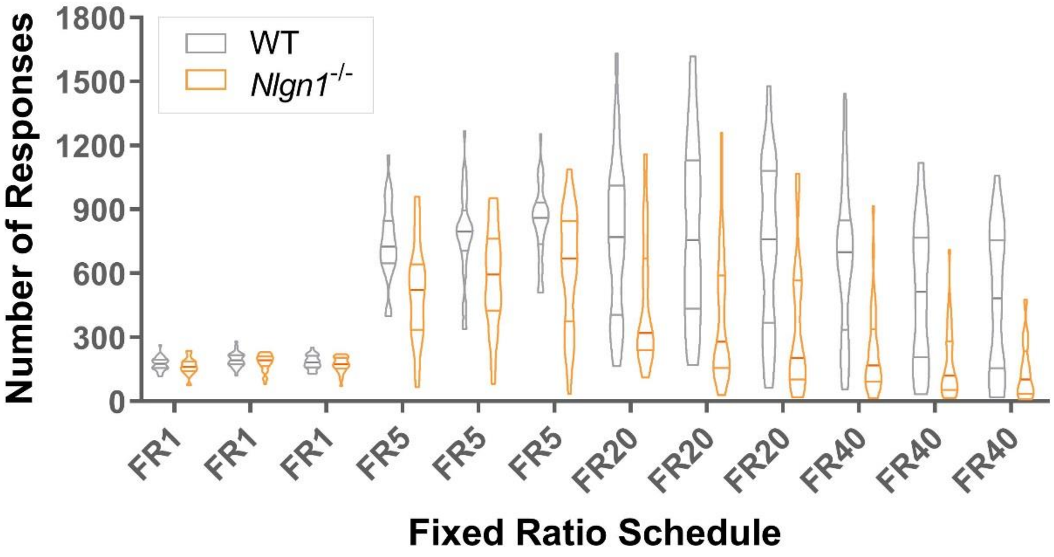

Figure S9: Number of responses made in fixed ratio task. *Nlgn1*$^{-/-}$ and wildtype (WT) mice tested on three sessions at each ratio requirement. Horizontal lines indicate 1st, 2nd (median) and 3rd quartiles.

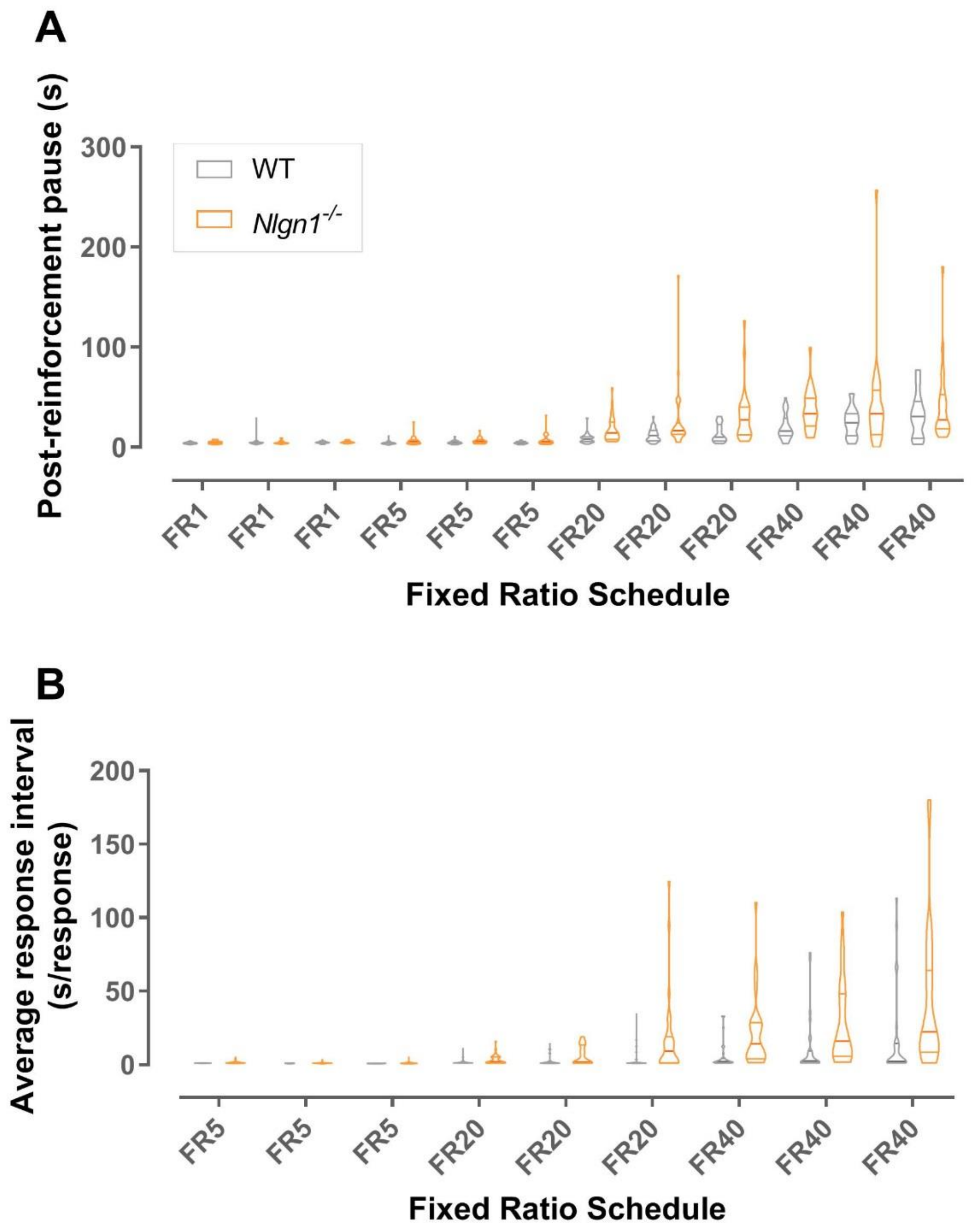

Figure S10: Post-reinforcement pause and average response interval in fixed ratio task. Three sessions at each ratio requirement. **(A)** Post-reinforcement pause: time to the first response after consuming a reward. Note data could not be gathered from animals that completed <1 trial. **(B)** Average response interval: time spent per response after the animal has made the first response of a trial. Horizontal lines indicate 1st, 2nd (median) and 3rd quartiles.

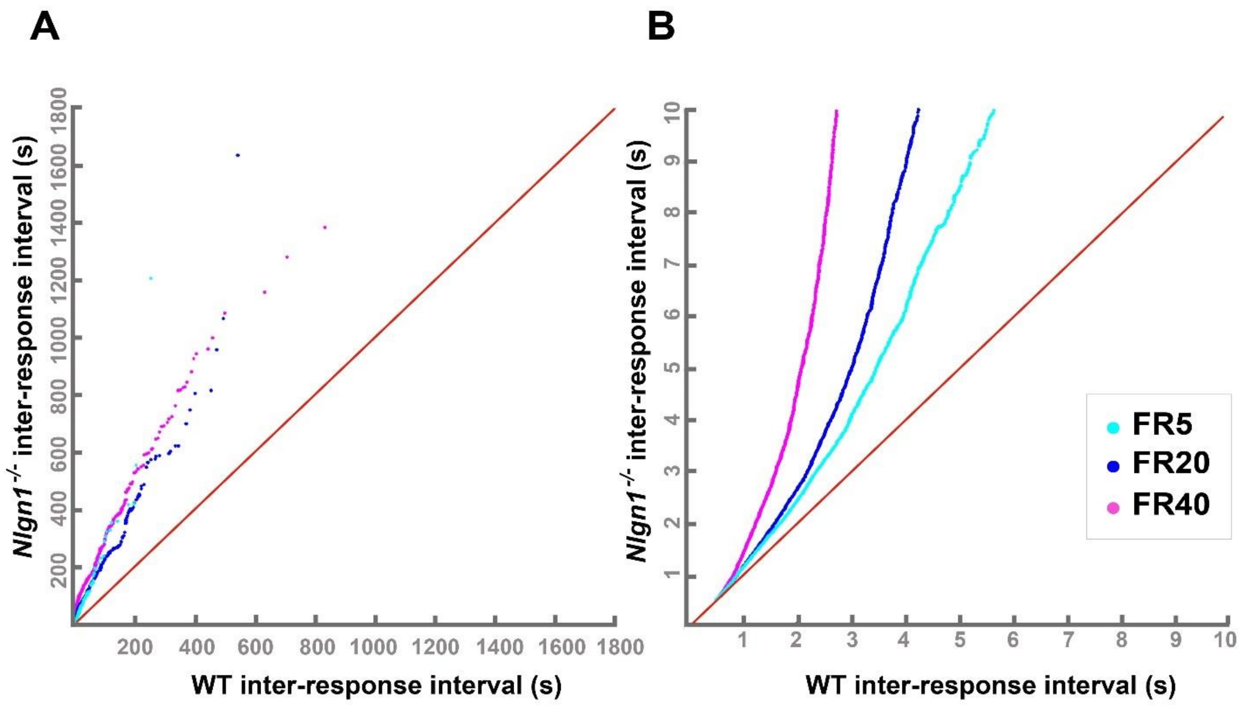

Figure S11: Response-by-response comparison of raw inter-response interval values between *Nlgn1*$^{-/-}$ and wildtype mice. Quantile-quantile (QQ) plots comparing the distribution of raw inter-response intervals (IRI) used to calculate the median session response intervals presented in Figure 4E. Wildtype (WT) IRI are on the X axis and *Nlgn1*$^{-/-}$ IRI on the Y axis. Red line indicates identical IRI at a given quantile. Shifts above the red line indicate longer IRI in *Nlgn1*$^{-/-}$ compared to WT mice in a given FR schedule at a given quantile. Notably, the shift becomes more pronounced as the response-reward ratio increases (FR40 > FR20 >FR5). **(A)** QQ plot covering the entire range of the dataset. **(B)** A close-up of (A) covering a smaller range.

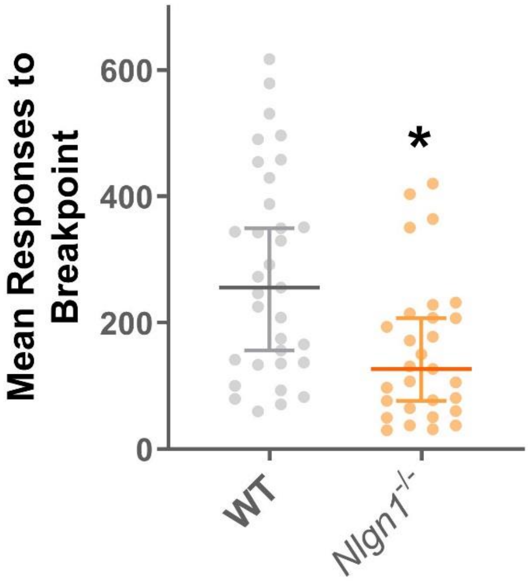

Figure S12: Average number of responses to breakpoint in progressive ratio task. *Nlgn1*$^{-/-}$ mice (naive cohort) made fewer responses than wildtype (WT) controls. Data from 6 sessions. Quantile regression (median), *$p < 0.05$, values represent median ± 95% CI.

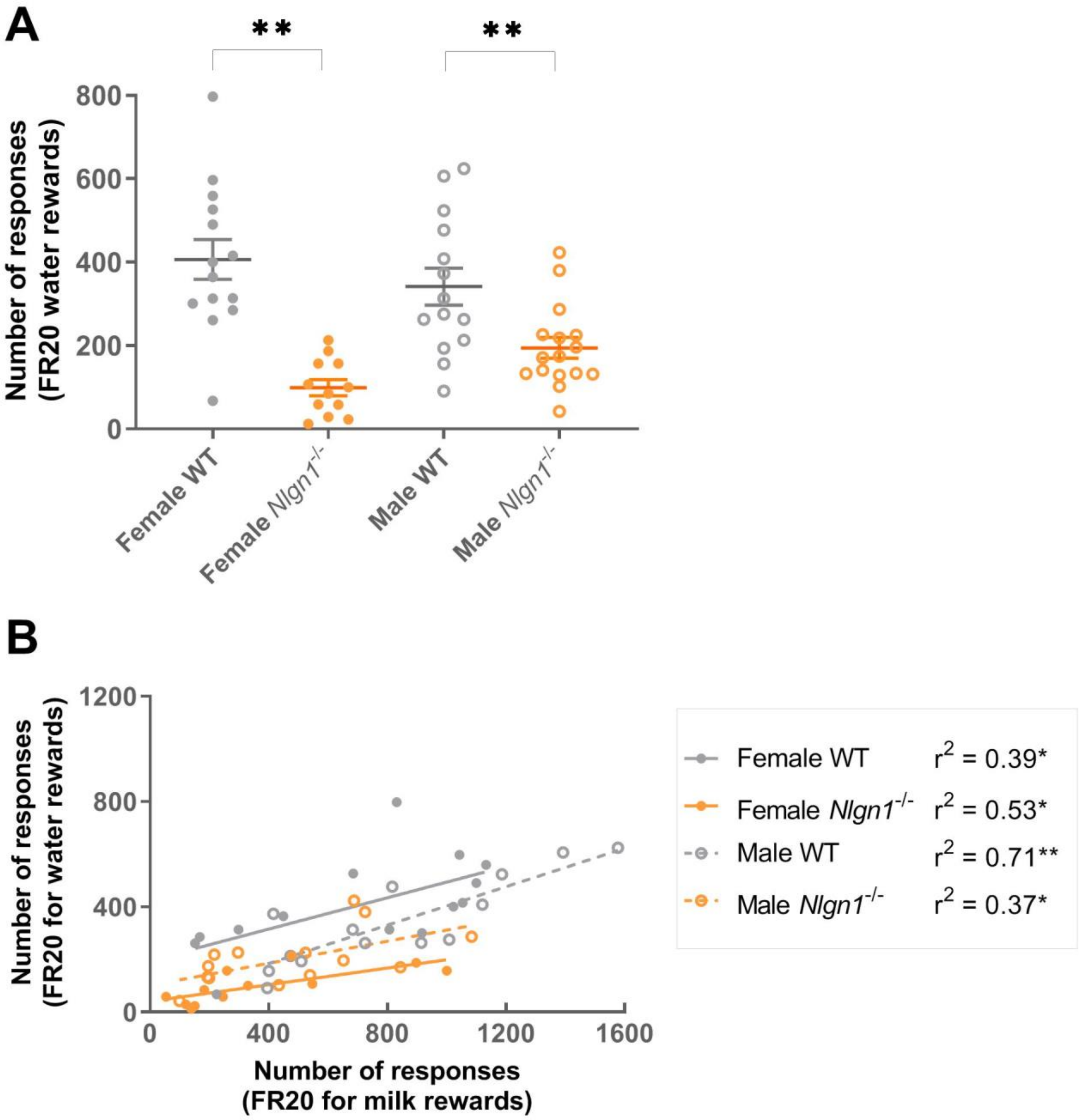

Figure S13: *Nlgn1$^{-/-}$* mice show decreased number of responses for water rewards. (A) Both male and female *Nlgn1$^{-/-}$* made significantly fewer responses than wildtype (WT) controls, but there was also a significant genotype x sex interaction with female *Nlgn1$^{-/-}$* mice making even less responses than male *Nlgn1$^{-/-}$* mice (see Additional File 2, Table S1 for statistics). **(B)** Positive correlations between number of responses mice made for milk and water rewards were significant for each sex and genotype group. Linear regression, *$p < 0.05$, **$p < 0.005$.

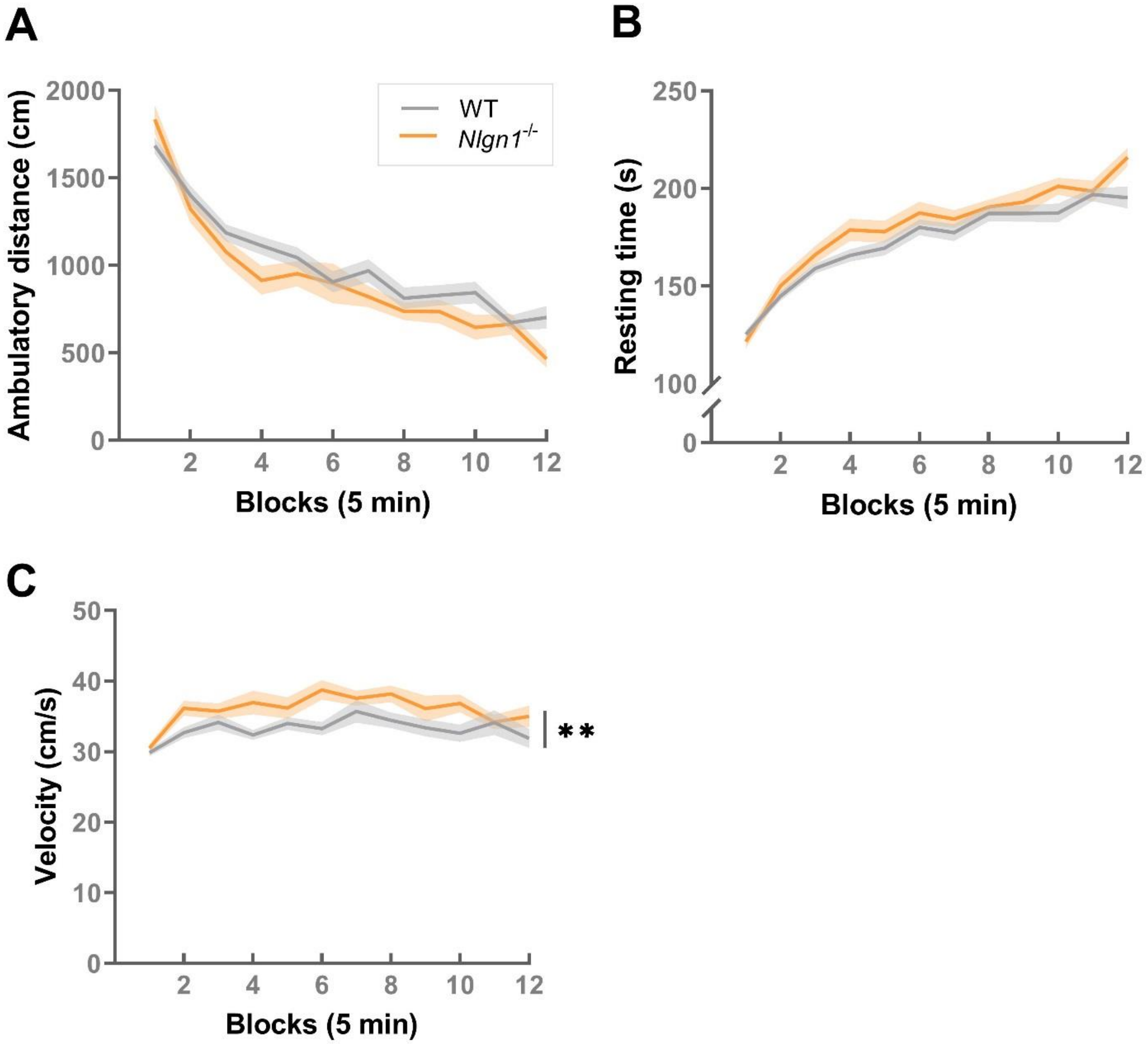

Figure S14: Experimentally naive *Nlgn1*$^{-/-}$ mice show subtle changes in exploration and spontaneous locomotor activity in a novel, open-field environment. (A) Ambulatory distance (centimeters) (generalized linear model) and **(B)** resting time (seconds) (mixed-effects linear model) showed no significant effect of genotype. **(C)** *Nlgn1*$^{-/-}$ mice also showed higher ambulatory velocity (centimeters/second) (effect of genotype $**p < 0.005$, mixed-effects linear model). Values represent means ± SEM.

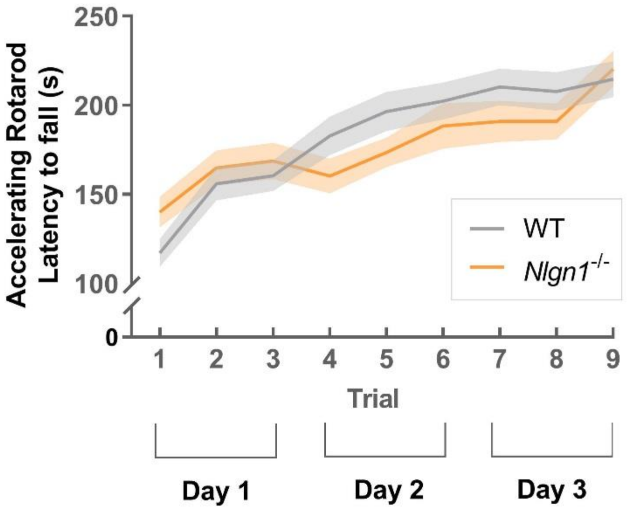

Figure S15: *Nlgn1$^{-/-}$* mice displayed normal motor coordination and learning on the accelerating rotarod test. No differences between genotypes in the latency to fall off the accelerating rotarod on a series of three 5-min trials tested across three consecutive days (9 trials in total). Linear regression, values represent means ± SEM.

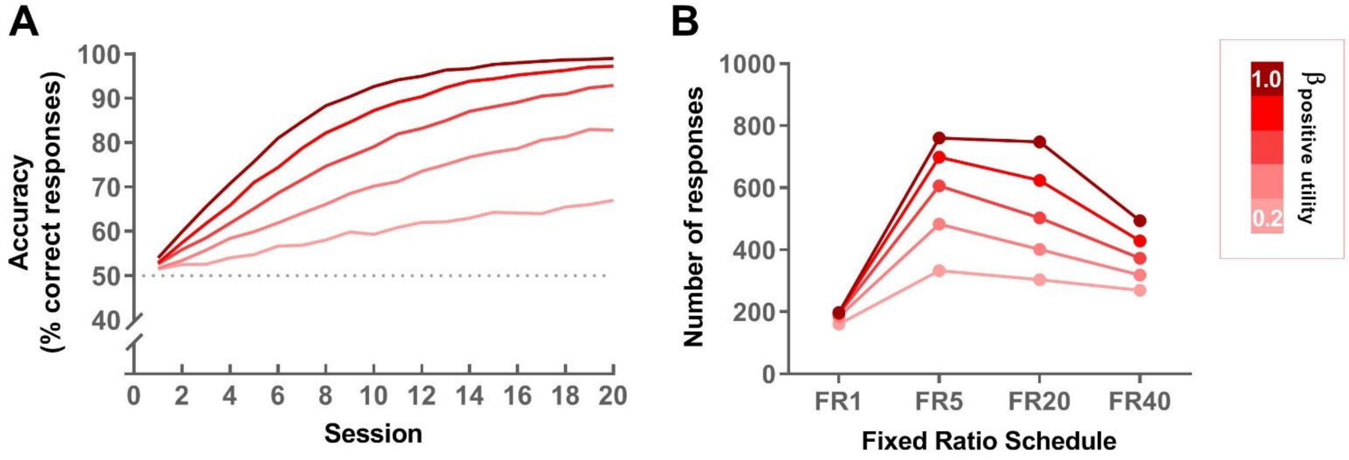

Figure S16: Simulated effect of decreasing the weighting on positive utilities (β_P) in the calculation of net utilities. (A) Decreasing β_P increases randomness in the choice between correct and incorrect responding in the simulated binary choice task leading to a flatter learning curve. **(B)** Decreasing β_p reduces number of responses in the simulated fixed ratio task.
