# Supplementary material for: A molecular insight into the dissociable regulation of associative learning and motivation by the synaptic protein neuroligin-1
Source: BMC Biol. 2020 Sep 14;18:118. doi: 10.1186/s12915-020-00848-7 (PMC7646379; doi:10.1186/s12915-020-00848-7)
Supplement: Supplementary file 2 — Additional file 2: Table S1. Variables included in regression models. [file 12915_2020_848_MOESM2_ESM.pdf]

Additional File 2

A molecular insight into the dissociable regulation of associative learning and motivation by the synaptic protein neuroligin-1

Jiaqi Luo[1], Jessica M Tan[1], Jess Nithianantharajah[1]

1. Florey Institute of Neuroscience and Mental Health, Florey Department of Neuroscience, Melbourne Brain Centre, University of Melbourne, Parkville Victoria Australia.

Table S1: Variables included in regression models

Dependent variable	Statistical model	Independent variables	Effect size	Lower CI.	Upper CI.	P value	Corresponding Figure
Trial outcome in PD (correct or incorrect)	Mixed-effect generalized linear model (logit link)	Genotype	0.94	0.81	1.10	0.47	Figure 1C
		Sex	1.07	0.92	1.25	0.38	
		Session	1.11	1.09	1.13	< 0.001	
		Trial within session	1.00	0.998	1.002	0.99	
		Stimulus location (right)	1.08	0.92	1.23	0.35	
		Genotype x Sex	0.98	0.72	1.34	0.88	
		Genotype x Session	0.97	0.93	1.00	0.064	
Trial outcome in PAL (correct or incorrect)	Mixed-effect generalized linear model (logit link)	Genotype	0.91	0.77	1.08	0.28	Figure 1F
		Sex	1.12	0.94	1.32	0.18	
		Session	1.03	1.03	1.04	< 0.001	
		Trial within session	1.00	0.999	1.001	0.922	
		Stimulus location (center)	0.46	0.39	0.54	< 0.001	
		Stimulus location (right)	1.07	0.85	1.34	0.58	
		Genotype x Sex	0.95	0.63	1.43	0.81	
		Genotype x Session	0.99	0.99	1.00	0.10	
Trial outcome in RL (correct or incorrect)	Mixed-effect generalized linear model (logit link)	Genotype	0.98	0.79	1.21	0.85	Figure 2C
		Sex	1.19	0.97	1.45	0.10	
		Session	1.09	1.08	1.11	< 0.001	
		Trial within session	1.00	0.998	1.001	0.29	
		Stimulus location (right)	1.07	0.88	1.30	0.51	
		Genotype x Sex	0.95	0.63	1.44	0.81	
		Genotype x Session	0.98	0.96	1.01	0.13	
Trial outcome in extinction learning (response or omission)	Mixed-effect generalized linear model (logit link)	Genotype	0.84	0.64	1.09	0.19	Figure 2F
		Sex	1.23	0.95	1.59	0.11	
		Session	0.53	0.50	0.57	< 0.001	
		Trial within session	0.93	0.92	0.94	< 0.001	
		Genotype x Sex	0.85	0.51	1.43	0.55	
		Genotype x Session	1.05	0.95	1.17	0.34	
		Genotype x Trial	1.00	0.99	1.02	0.68	
Trial outcome in PD (correct or incorrect)	Mixed-effect generalized linear model (logit link)	Genotype	0.97	0.85	1.09	0.57	Figure 2G
		Sex	1.02	0.90	1.16	0.74	
		Session	1.10	1.08	1.12	<0.001	
		Trial within session	1.000	0.999	1.002	0.70	
		Stimulus location (right)	1.09	0.94	1.26	0.28	
		Correction trial	0.56	0.52	0.61	<0.001	
		Genotype x Correction trial	1.06	0.89	1.25	0.53	
Trial outcome in PAL (correct or incorrect)	Mixed-effect generalized linear model (logit link)	Genotype	0.91	0.80	1.04	0.17	Figure 2G
		Sex	1.06	0.94	1.21	0.35	
		Session	1.04	1.03	1.04	< 0.001	
		Trial within session	0.999	0.998	1.000	0.06	
		Stimulus location (center)	0.50	0.44	0.57	< 0.001	
		Stimulus location (right)	1.11	0.94	1.31	0.21	
		Correction trial	0.73	0.68	0.77	< 0.001	
		Genotype x Correction trial	1.00	0.87	1.14	0.95	
Trial outcome in RL (correct or incorrect)	Mixed-effect generalized linear model (logit link)	Genotype	0.94	0.81	1.10	0.43	Figure 2G
		Sex	1.16	1.00	1.34	0.04	
		Session	1.10	1.09	1.11	< 0.001	
		Trial within session	0.999	0.998	1.000	0.24	
		Stimulus location (right)	1.03	0.90	1.17	0.70	
		Correction trial	0.75	0.70	0.80	< 0.001	
		Genotype x Correction trial	0.95	0.81	1.10	0.48	

Trial outcome in PD (correct or incorrect)	Mixed-effect generalized linear model (logit link)	Genotype	0.94	0.81	1.10	0.47	Figure 2G
		Sex	1.07	0.92	1.25	0.37	
		Session	1.11	1.09	1.13	< 0.001	
		Trial within session	1.00	0.998	1.002	0.92	
		Stimulus location (right)	1.08	0.92	1.28	0.34	
		Reoccurring trial	1.12	1.04	1.21	< 0.001	
		Genotype x Reoccurring trial	1.12	0.97	1.31	0.13	
Trial outcome in PAL (correct or incorrect)	Mixed-effect generalized linear model (logit link)	Genotype	0.91	0.77	1.08	0.28	Figure 2G
		Sex	1.12	0.95	1.32	0.18	
		Session	1.03	1.03	1.04	< 0.001	
		Trial within session	1.000	0.999	1.001	1.00	
		Stimulus location (center)	0.46	0.39	0.54	< 0.001	
		Stimulus location (right)	1.07	0.85	1.34	0.58	
		Reoccurring trial	1.20	1.13	1.28	< 0.001	
		Genotype x Reoccurring trial	0.97	0.85	1.11	0.64	
Trial outcome in RL (correct or incorrect)	Mixed-effect generalized linear model (logit link)	Genotype	0.98	0.79	1.21	0.86	Figure 2G
		Sex	1.18	0.97	1.45	0.10	
		Session	1.10	1.08	1.11	< 0.001	
		Trial within session	0.999	0.998	1.000	0.19	
		Stimulus location (right)	1.07	0.88	1.30	0.51	
		Reoccurring trial	1.21	1.14	1.29	<0.001	
		Genotype x Reoccurring trial	0.96	0.85	1.08	0.49	
Initiation latency in PD	Quantile regression (median)	Genotype	1.13	0.48	1.77	0.001	Figure 3B
		Sex	-0.12	-0.75	0.52	0.72	
		Session	-0.35	-0.43	-0.28	< 0.001	
		Trial within session	0.02	0.01	0.03	< 0.001	
		Stimulus location (right)	-0.07	-0.23	0.08	0.35	
		Correction trial	-1.25	-1.57	-0.93	< 0.001	
		Genotype x Sex	-0.19	-1.44	1.06	0.77	
Initiation latency in RL	Quantile regression (median)	Genotype	0.87	0.36	1.37	0.001	Figure 3B
		Sex	0.16	-0.25	0.57	0.45	
		Session	-0.14	-0.17	-0.12	<.001	
		Trial within session	0.020	0.015	0.024	< 0.001	
		Stimulus location (right)	-0.02	-0.11	0.07	0.639	
		Correction trial	-0.31	-0.48	-0.15	< 0.001	
		Genotype x Sex	0.17	-0.82	1.17	0.734	
Initiation latency in PAL	Quantile regression (median)	Genotype	0.42	0.04	0.79	0.03	Figure 3B
		Sex	0.03	-0.27	0.33	0.839	
		Session	-0.025	-0.030	-0.021	< 0.001	
		Trial within session	0.016	0.012	0.019	< 0.001	
		Stimulus location (center)	0.05	0.00	0.10	0.063	
		Stimulus location (right)	-0.01	-0.06	0.04	0.61	
		Correction trial	-0.17	-0.30	-0.03	0.013	
		Genotype x Sex	-0.14	-0.89	0.61	0.714	
Reward collection latency in PD	Quantile regression (median	Genotype	0.13	0.07	0.19	< 0.001	Figure 3C
		Sex	0.02	-0.04	0.09	0.44	
		Session	-0.012	-0.016	-0.009	< 0.001	
		Trial within session	-0.0006	-0.0011	-0.0001	0.02	
		Stimulus location (right)	-0.01	-0.04	0.01	0.34	
		Correction trial	-0.02	-0.03	-0.01	< 0.001	
		Genotype x Sex	0.01	-0.03	0.04	0.71	
Reward collection latency in RL	Quantile regression (median	Genotype	0.13	0.07	0.19	< 0.001	Figure 3C
		Sex	0.00	-0.06	0.05	0.89	
		Session	-0.002	-0.004	0.000	0.12	
		Trial within session	-0.0008	-0.0013	-0.0004	< 0.001	
		Stimulus location (right)	0.01	-0.01	0.04	0.31	
		Correction trial	-0.01	-0.03	0.00	0.02	
		Genotype x Sex	-0.01	-0.14	0.12	0.90	

Reward collection latency in PAL	Quantile regression (median	Genotype	0.07	0.02	0.13	0.01	Figure 3C
		Sex	0.02	-0.04	0.08	0.53	
		Session	0.000	-0.001	0.002	0.54	
		Trial within session	-0.0001	-0.0005	0.0003	0.74	
		Stimulus location (center)	-0.05	-0.08	-0.03	< 0.001	
		Stimulus location (right)	-0.02	-0.05	0.02	0.41	
		Correction trial	-0.02	-0.03	-0.01	< 0.001	
		Genotype x Sex	0.02	-0.10	0.13	0.79	
Stimulus-approach latency in PD	Quantile regression (median)	Genotype	0.06	-0.02	0.13	0.14	Figure 3D
		Sex	-0.05	-0.13	0.02	0.16	
		Session	-0.032	-0.038	-0.026	< 0.001	
		Trial within session	-0.0007	-0.0014	0	0.06	
		Stimulus location (right)	0.00	-0.02	0.01	0.59	
		Correction trial	0.05	0.03	0.07	< 0.001	
		Genotype x Sex	-0.04	-0.18	0.11	0.63	
Stimulus-approach latency in RL	Quantile regression (median)	Genotype	0.07	-0.01	0.15	0.08	Figure 3D
		Sex	-0.01	-0.09	0.06	0.71	
		Session	-0.02	-0.02	-0.02	< 0.001	
		Trial within session	-0.0002	-0.0010	0.0005	0.53	
		Stimulus location (right)	0.00	-0.01	0.02	0.67	
		Correction trial	0.02	0.00	0.04	0.02	
		Genotype x Sex	-0.02	-0.19	0.14	0.77	
Stimulus-approach latency in PAL	Quantile regression (median)	Genotype	0.01	-0.07	0.09	0.74	Figure 3D
		Sex	-0.06	-0.14	0.02	0.18	
		Session	-0.002	-0.003	-0.001	0.002	
		Trial within session	0.0010	0.0005	0.0015	< 0.001	
		Stimulus location (center)	0.03	0.02	0.04	< 0.001	
		Stimulus location (right)	-0.01	-0.02	0.00	0.20	
		Correction trial	0.03	0.02	0.05	< 0.001	
		Genotype x Sex	-0.03	-0.20	0.14	0.72	
Stimulus-selection latency in PD	Quantile regression (median)	Genotype	-0.07	-0.27	0.13	0.51	Figure 3E
		Sex	-0.04	-0.24	0.16	0.70	
		Session	-0.02	-0.03	0.00	0.04	
		Trial within session	-0.002	-0.004	-0.001	0.01	
		Stimulus location (right)	0.02	-0.09	0.12	0.75	
		Correction trial	-0.15	-0.21	-0.09	< 0.001	
		Genotype x Sex	0.31	-0.07	0.70	0.11	
Stimulus-selection latency in RL	Quantile regression (median)	Genotype	-0.03	-0.25	0.19	0.80	Figure 3E
		Sex	0.04	-0.17	0.25	0.73	
		Session	-0.02	-0.03	-0.01	0.000	
		Trial within session	0.0010	-0.0006	0.0026	0.23	
		Stimulus location (right)	0.039	-0.036	0.113	0.31	
		Correction trial	-0.05	-0.09	-0.02	0.004	
		Genotype x Sex	0.20	-0.22	0.62	0.36	
Stimulus-selection latency in PAL	Quantile regression (median)	Genotype	0.00	-0.18	0.17	0.96	Figure 3E
		Sex	0.06	-0.11	0.23	0.46	
		Session	0.005	0.002	0.009	0.01	
		Trial within session	-0.0003	-0.0014	0.0007	0.52	
		Stimulus location (center)	0.29	0.20	0.39	< 0.001	
		Stimulus location (right)	-0.26	-0.41	-0.12	< 0.001	
		Correction trial	0.01	-0.03	0.06	0.53	
		Genotype x Sex	0.10	-0.24	0.45	0.56	
Total number of responses FR1	Quantile regression (median)	Genotype	-7	-27.30	13.30	0.50	Figure 4B - C
		Sex	20	-0.26	40.26	0.053	
		Session	2	-4.00	8.00	0.51	
		Genotype x Sex	8	-31.16	47.16	0.69	

Total number of responses FR5	Quantile regression (median)	Genotype	-215	-342.62	-87.38	< 0.001	Figure 4B - C
		Sex	55	-62.15	172.15	0.36	
		Session	80	41.82	118.18	< 0.001	
		Genotype x Sex	127.5	-120.71	375.71	0.31	
Total number of responses FR20	Quantile regression (median)	Genotype	-491	-723.19	-258.81	< 0.001	Figure 4B - C
		Sex	81	-119.91	281.91	0.43	
		Session	-49	-87.32	-10.68	0.01	
		Genotype x Sex	-36	-510.65	438.65	0.88	
Total number of responses FR40	Quantile regression (median)	Genotype	-442	-617.02	-266.98	< 0.001	Figure 4B - C
		Sex	-20	-149.25	109.25	0.76	
		Session	-58	-84.51	-31.49	< 0.001	
		Genotype x Sex	223	-106.71	552.71	0.18	
Total number of responses FR20 (water rewards)	Mixed-effect linear regression	Genotype	-230.50	-312.36	-148.64	< 0.001	Figure 4F Figure S13A
		Sex	16.51	-54.72	87.74	0.65	
		Session	-12.73	-28.75	3.30	0.12	
		Water-restricted weight	4.01	-7.84	15.87	0.51	
		Genotype x Sex	155.27	19.24	291.30	0.03	
		Genotype (female)	-312.65	-427.37	-197.93	< 0.001	
		Genotype (male)	-166.66	-275.64	-57.68	0.003	
Correlation between responses for water and milk rewards	Linear regression	Responses for milk (WT)	0.31	0.17	0.45	< 0.001	Figure 4G Figure S13B
		Responses for milk (*Nlgn1*$^{-/-}$)	0.20	0.10	0.31	0.001	
		Responses for milk (female WT)	0.31	0.06	0.55	0.018	
		Responses for milk (male WT)	0.36	0.22	0.51	0.000	
		Responses for milk (female *Nlgn1*$^{-/-}$)	0.16	0.05	0.26	0.007	
		Responses for milk (male *Nlgn1*$^{-/-}$)	0.21	0.05	0.37	0.012	
Ambulatory distance spontaneous locomotor activity in open field (operant experience cohort)	Mixed-effect generalized linear model (log link)	Genotype	0.78	0.71	0.86	< 0.001	Figure 5C
		Sex	0.91	0.82	1.00	0.06	
		Time (5-minute block)	0.95	0.94	0.96	< 0.001	
		Cohort	1.01	0.90	1.12	0.88	
		Genotype x Sex	1.12	0.91	1.38	0.27	
		Genotype x Time	0.98	0.96	1.00	0.06	
		Genotype x Cohort	0.94	0.75	1.18	0.60	
Resting time spontaneous locomotor activity in open field (operant experience cohort)	Mixed-effect linear regression	Genotype	11.78	5.34	18.22	< 0.001	Figure 5D
		Sex	5.18	-1.40	11.75	0.12	
		Time (5-minute block)	4.26	3.73	4.79	< 0.001	
		Cohort	-0.84	-7.89	6.21	0.82	
		Genotype x Sex	-4.54	-17.81	8.73	0.50	
		Genotype x Time	0.58	-0.47	1.64	0.28	
		Genotype x Cohort	0.89	-13.53	15.30	0.90	
Ambulatory velocity spontaneous locomotor activity in open field (operant experience cohort)	Mixed-effect linear regression	Genotype	0.38	-0.71	1.48	0.49	Figure 5E
		Sex	-0.32	-1.43	0.78	0.56	
		Time (5-minute block)	-0.27	-1.43	0.89	0.65	
		Cohort	0.40	0.28	0.53	< 0.001	
		Genotype x Sex	1.49	-0.79	3.77	0.20	
		Genotype x Time	-0.22	-0.48	0.04	0.09	
		Genotype x Cohort	-0.44	-2.68	1.79	0.70	
Total mobility time in Porsolt swim test	Linear regression	Genotype	35.81	14.12	57.51	< 0.001	Figure 5F
		Sex	5.58	-16.12	27.27	0.61	
		Genotype x Sex	5.16	-38.76	49.09	0.81	
Trial outcome in PD (correct or incorrect)	Mixed-effect generalized linear model (logit link)	Genotype	0.96	0.85	1.08	0.49	Figure S7
		Sex	1.03	0.91	1.17	0.62	
		Session	1.10	1.08	1.13	< 0.001	
		Trial within session	1.000	0.999	1.002	0.60	
		Stimulus location (right)	1.08	0.93	1.26	0.30	
		Stimulus-selection latency	1.07	1.03	1.10	< 0.001	
		Correction trial	0.56	0.52	0.61	< 0.001	

Trial outcome in PAL (correct or incorrect)	Mixed-effect generalized linear model (logit link)	Genotype	0.91	0.80	1.04	0.16	Figure S7
		Sex	1.06	0.94	1.20	0.34	
		Session	1.038	1.034	1.042	< 0.001	
		Trial within session	0.999	0.998	1.000	0.04	
		Stimulus location (center)	0.49	0.43	0.56	< 0.001	
		Stimulus location (right)	1.13	0.95	1.33	0.17	
		Stimulus-selection latency	1.07	1.04	1.10	< 0.001	
		Correction trial	0.72	0.68	0.77	< 0.001	
Trial outcome in RL (correct or incorrect)	Mixed-effect generalized linear model (logit link)	Genotype	0.95	0.83	1.08	0.40	Figure S7
		Sex	1.16	1.02	1.32	0.02	
		Session	1.10	1.09	1.12	< 0.001	
		Trial within session	0.999	0.998	1.000	0.17	
		Stimulus location (right)	1.02	0.90	1.17	0.73	
		Stimulus-selection latency	1.04	1.01	1.06	0.01	
		Correction trial	0.74	0.70	0.79	< 0.001	
Trial outcome in PD (correct or incorrect)	Mixed-effect generalized linear model (logit link)	Genotype	0.97	0.85	1.10	0.59	Figure S7
		Sex	1.02	0.90	1.16	0.76	
		Session	1.10	1.08	1.12	< 0.001	
		Trial within session	1.000	0.999	1.002	0.73	
		Stimulus location (right)	1.09	0.94	1.26	0.28	
		Stimulus-approach latency	1.00	0.98	1.01	0.54	
		Correction trial	0.56	0.52	0.61	< 0.001	
Trial outcome in PAL (correct or incorrect)	Mixed-effect generalized linear model (logit link)	Genotype	0.91	0.80	1.04	0.18	Figure S7
		Sex	1.06	0.93	1.20	0.38	
		Session	1.038	1.034	1.042	< 0.001	
		Trial within session	0.999	0.998	1.000	0.08	
		Stimulus location (center)	0.50	0.44	0.57	< 0.001	
		Stimulus location (right)	1.11	0.94	1.31	0.22	
		Stimulus-approach latency	0.99	0.98	1.00	0.01	
		Correction trial	0.73	0.68	0.78	< 0.001	
Trial outcome in RL (correct or incorrect)	Mixed-effect generalized linear model (logit link)	Genotype	0.94	0.80	1.11	0.47	Figure S7
		Sex	1.16	0.99	1.35	0.06	
		Session	1.10	1.09	1.11	< 0.001	
		Trial within session	0.999	0.998	1.000	0.22	
		Stimulus location (right)	1.03	0.90	1.17	0.70	
		Stimulus-approach latency	0.99	0.98	1.00	0.13	
		Correction trial	0.75	0.70	0.80	< 0.001	
Total number of responses progressive ratio	Quantile regression (median)	Genotype	-112.60	-191.89	-33.31	0.01	Figure S12
		Sex	20.00	-57.86	97.86	0.61	
		Session	-9.40	-19.13	0.33	0.06	
		Cohort	-96.60	-172.44	-20.76	0.01	
		Genotype x Sex	-8.40	-175.99	159.19	0.92	
		Genotype x Cohort	127.75	-8.84	264.34	0.07	
Ambulatory distance spontaneous locomotor activity in open field (experimentally naive cohort)	Mixed-effect generalized linear model (log link)	Genotype	0.93	0.82	1.05	0.24	Figure S14A
		Sex	0.92	0.81	1.04	0.17	
		Time (5-minute block)	0.91	0.90	0.92	< 0.001	
		Genotype x Sex	1.15	0.89	1.49	0.28	
		Genotype x Time	0.97	0.95	0.99	0.01	
		Time (WT)	0.925	0.915	0.934	< 0.001	
		Time (*Nlgn1*$^{-/-}$)	0.90	0.88	0.92	< 0.001	
Resting time spontaneous locomotor activity in open field (experimentally naive cohort)	Mixed-effect linear regression	Genotype	7.32	-0.78	15.42	0.08	Figure S14B
		Sex	3.66	-4.25	11.57	0.36	
		Time (5-minute block)	5.96	5.46	6.47	< 0.001	
		Genotype x Sex	-12.89	-28.67	2.89	0.11	
		Genotype x Time	0.81	-0.20	1.81	0.11	

Ambulatory velocity spontaneous locomotor activity in open field (experimentally naive cohort)	Mixed-effect linear regression	Genotype	2.97	1.27	4.67	0.001	Figure S14C
		Sex	-0.13	-1.89	1.63	0.89	
		Time (5-minute block)	0.15	0.01	0.29	0.04	
		Genotype x Sex	0.49	-3.04	4.02	0.79	
		Genotype x Time	0.07	-0.21	0.35	0.64	
Latency to fall accelerating rotarod	Mixed-effect linear regression	Session	9.58	7.93	11.23	< 0.001	Figure S15
		Genotype	-8.31	-28.90	12.29	0.43	
		Sex	12.39	-8.02	32.80	0.23	
		Cohort	46.96	26.97	66.95	< 0.001	
		Genotype x Sex	3.45	-38.26	45.16	0.87	
		Genotype x Cohort	1.86	-39.16	42.88	0.93	
